# Diversity from genes to ecosystems: A unifying framework to study variation across biological metrics and scales

**DOI:** 10.1111/eva.12593

**Published:** 2018-02-20

**Authors:** Oscar E. Gaggiotti, Anne Chao, Pedro Peres‐Neto, Chun‐Huo Chiu, Christine Edwards, Marie‐Josée Fortin, Lou Jost, Christopher M. Richards, Kimberly A. Selkoe

**Affiliations:** ^1^ School of Biology Scottish Oceans Institute University of St Andrews St Andrews UK; ^2^ Institute of Statistics National Tsing Hua University Hsin‐Chu Taiwan; ^3^ Department of Biology Concordia University Montreal QC Canada; ^4^ Department of Agronomy National Taiwan University Taipei Taiwan; ^5^ Center for Conservation and Sustainable Development Missouri Botanical Garden Saint Louis MO USA; ^6^ Department of Ecology and Evolutionary Biology University of Toronto Toronto ON Canada; ^7^ Ecominga Fundation Banos Tungurahua Ecuador; ^8^ Plant Germplasm Preservation Research Unit USDA‐ARS Fort Collins CO USA; ^9^ National Center for Ecological Analysis and Synthesis University of California Santa Barbara Santa Barbara CA USA; ^10^ Hawai'i Institute of Marine Biology University of Hawai'i at Mānoa Kaneohe HI USA

**Keywords:** biodiversity indices, genetic diversity, hierarchical spatial structure, Hill numbers, species diversity

## Abstract

Biological diversity is a key concept in the life sciences and plays a fundamental role in many ecological and evolutionary processes. Although biodiversity is inherently a hierarchical concept covering different levels of organization (genes, population, species, ecological communities and ecosystems), a diversity index that behaves consistently across these different levels has so far been lacking, hindering the development of truly integrative biodiversity studies. To fill this important knowledge gap, we present a unifying framework for the measurement of biodiversity across hierarchical levels of organization. Our weighted, information‐based decomposition framework is based on a Hill number of order *q* = 1, which weights all elements in proportion to their frequency and leads to diversity measures based on Shannon's entropy. We investigated the numerical behaviour of our approach with simulations and showed that it can accurately describe complex spatial hierarchical structures. To demonstrate the intuitive and straightforward interpretation of our diversity measures in terms of effective number of components (alleles, species, etc.), we applied the framework to a real data set on coral reef biodiversity. We expect our framework will have multiple applications covering the fields of conservation biology, community genetics and eco‐evolutionary dynamics.

## 1 INTRODUCTION

Biological diversity is a foundational concept in the life sciences and critical to strategies for ecological conservation. However, for many decades, biodiversity has been treated in a piecemeal manner with ecologists focusing on species diversity (but more recently also on trait and phylogenetic diversity) and population geneticists focusing on genetic diversity. This dichotomy has led to large differences in the type of diversity indices that have been used to measure species, trait, phylogenetic and genetic diversity. Ecologists were initially focused on empirical developments and generated a very large number of species diversity indices that strongly differ in their numerical behaviour (Jost, 2006) and estimation properties (Bunge, Willis, & Walsh, 2014). On the other hand, population genetics was initially dominated by theoretical developments and mathematical models focused on a specific set of parameters that described genetic diversity within and among populations, which led to the development of a restricted set of genetic diversity indices. Thus, although biodiversity is inherently a hierarchical concept covering different levels of organization (genetic, population, species, ecological communities and ecosystems), the lack of diversity indices that behave consistently across these different levels has precluded the development of truly integrative biodiversity studies.

Recently, motivated by this lack of common measures for biodiversity at different levels of biological organization, population geneticists have carried out methodological developments that extend the use of popular species diversity indices to the measurement genetic diversity at different levels of spatial subdivision [e.g., Shannon's and Simpson's indices (Sherwin, Jabot, Rush, & Rossetto, 2006; Smouse, Whitehead, & Peakall, 2015)]. However, simply adapting species diversity measures is not sufficient for two reasons. First, there is much controversy over how to quantify abundance‐based species diversity in a community (Mendes, Evangelista, Thomaz, Agostinho, & Gomes, 2008). Second, there has been little agreement on how to partition diversity into its spatial components (Ellison, 2010). A promising solution for a unified measure of genetic diversity centres on Hill numbers (Hill, 1973). Indeed, a consensus is emerging on the use of Hill numbers as a unifying concept to define measures of various types of diversity including species, phylogenetic and functional diversities (Chao, Chiu, & Jost, 2014). Importantly, Hill numbers follow the replication principle, ensuring that diversity measures are linear in relation to group pooling. As such, they can be used to develop proper partition schemes across spatial scales or other hierarchical structures such as populations within metapopulations, species within phylogenies, communities within ecosystems and to pool information across different levels in a hierarchy.

The purpose of this study was to present a unifying framework for the measurement of biodiversity across hierarchical levels of organization, from local population to ecosystem levels. We expect that this new framework will be a useful tool for conservation biologists and will also facilitate the development of the fields of community genetics (Agrawal, 2003) and eco‐evolutionary dynamics (Hendry, 2013). This new framework may also facilitate bridging community ecology processes (selection among species, drift, dispersal and speciation) and the processes emphasized by population genetics theory (selection within species, drift, gene flow and mutation) as explored by Vellend et al. (2014). The paper starts by outlining historical developments on the formulation and use of biodiversity measures in the fields of ecology and population genetics (Section 2). We then provide an overview of the use of Hill numbers in ecology and their relationship with population genetic parameters such as *N*_*e*_ (Section 3). Section 4 presents a weighted information‐based decomposition framework that provides measures of both genetic and species diversity at all hierarchical levels of spatial subdivision, from populations to ecosystems. This is followed by the description of software that implements the approach (Section 5). Section 6 explores patterns of species and genetic diversity under different spatial subdivision models using simulated data with known diversity hierarchical structures. Section 7 shows an application to a real data set on coral reef biodiversity (Selkoe et al., 2016). We close with a discussion of the advantages and limitations of our approach and its applications in the fields of conservation biology, community genetics and eco‐evolutionary dynamics.

## 2 HISTORICAL DEVELOPMENTS

Arguably, the ultimate reason for methodological divergence in diversity indices used by population geneticists and community ecologists resides in the very different contexts that lead to the emergence of these two disciplines. Ecologists were interested in understanding the processes that determine the structure and composition of communities and could directly measure the community traits (number of species and their abundances) needed to compare different communities. This relatively easy access to real data and an initially limited interest in mechanistic models fostered the development of a large number of diversity measures to explore species distributional data (Magurran, 2004) and eventually made the quantification of abundance‐based species diversity, one of the most controversial issues in ecology. Population genetics, on the other hand, arose in response to a need to reconcile two opposing views of evolution that hinged on the type of diversity upon which natural selection acted. Darwin proposed that it was small continuous variation while Galton believed that natural selection acted upon large discontinuous variation (Provine, 1971). Variation in this case was an abstract concept and could not be directly measured, which motivated the development of a vast body of theory centred around mathematical models describing the behaviour of a restricted set of diversity measures (Provine, 1971).

Although ecologists and population geneticists use very different approaches to measure diversity, they are both interested in describing spatial patterns by decomposing total diversity into within‐ and among‐community/population components. But here again, methodological developments differ greatly between the two disciplines. Ecologists engaged in intense debates on the choice of partitioning schemes (Jost, 2007) while population geneticists remained largely faithful to the use of so‐called fixation indices proposed by Wright (1951). Nevertheless, the recently established fields of molecular ecology, community genetics and eco‐evolutionary dynamics are helping to foster a convergence between the methods used to measure species and genetic diversity. Indeed, in the last decade, population geneticists have begun to extend the use of popular species diversity metrics to the measurement of genetic diversity by deriving mathematical expressions linking them with evolutionary parameters such as effective population size and mutation and migration rates (Chao et al., 2015; Sherwin, 2010; Sherwin et al., 2006; Smouse et al., 2015).

Regardless of this very recent methodological convergence, ecologists and population geneticists face the same challenges when trying to characterize how diversity components (alpha, beta) are structured geographically. These problems have been described in great detail in the literature (e.g., see Jost, 2007, 2010), so here we will only give a very brief summary. The first problem is that the commonly used within‐community and within‐population abundance diversity measures (e.g., Shannon‐Wiener index and heterozygosity) are in fact entropies, meaning that they quantify the uncertainty in the species or allele identity of randomly sampled individuals or alleles, respectively. Importantly, these indices do not scale linearly with an increase in diversity and some of them (e.g., heterozygosity) reach an asymptote for large values. The second problem is that the “within‐” (alpha) and “between‐” (beta) components of diversity are not independent. Intuitively, if beta depends on alpha, it would be impossible to compare beta diversities across all levels at which alpha diversities differ.

Partitioning components of diversity is central to progress on these problems. Ecologists have related the traditional alpha, beta and gamma diversity using both additive and multiplicative schemes of partitioning. On the other hand, population geneticists have always used the multiplicative scheme based on the partitioning of the probability of identity by descent of pairs of alleles (inbreeding coefficients, *F*). Although there has been some confusion (cf. Jost, 2008; Jost et al., 2010; Meirmans & Hedrick, 2011), it is easy to demonstrate that all estimators of *F*_ST_, a parameter that quantifies genetic structure, including *G*_ST_ (Nei 1973) and θ (Weir & Cockerham, 1984), are based on the well‐known multiplicative decomposition of Wright's (1951) *F*‐statistics: $\left(1-F_{\mathrm{IT}}\right)=\left(1-F_{\mathrm{IS}}\right)\left(1-F_{\mathrm{ST}}\right)$, where all terms are entropy measures describing the uncertainty in the identity by descent of pairs of alleles, when they are sampled from the whole set of populations (metapopulation) $\left(1-F_{\mathrm{IT}}\right)$, from within the same population $\left(1-F_{\mathrm{IS}}\right)$, or from two different populations $\left(1-F_{\mathrm{ST}}\right)$.

As mentioned earlier, ecologists engaged in intense debates on how to partition species diversity but in a recent Ecology forum (Ellison, 2010), contributors agreed that a first step towards reaching a consensus was to adopt Hill numbers to measure diversity. Discussions among population geneticists are less advanced because of their traditional focus on the use of genetic polymorphism data to estimate important evolutionary parameters, which requires that genetic diversity statistics be effective measures of the causes and consequences of genetic differentiation (e.g., Whitlock, 2011). Much theoretical work is still needed to demonstrate that diversity measures based on information theory do satisfy this requirement. Here, instead, we argue that the adoption of Hill numbers in population genetics is also a good starting point to reach a consensus on how to partition genetic diversity. In what follows, we first introduce Hill numbers and then present a weighted information‐based decomposition framework applicable to both community and population genetics studies.

## 3 OVERVIEW OF HILL NUMBERS

There are now many articles describing the application of Hill numbers. Here, we follow Jost (2006), who reintroduced their use in ecology. As Jost (2006) noted, most diversity indices are in fact entropies that measure the uncertainty in the identity of species (or alleles) in a sample. However, true diversity measures should provide estimates of the number of distinct elements (species or alleles) in an aggregate (community or population). To derive such measures, we first note that diversity indices create equivalence classes among aggregates in the sense that all aggregates with the same diversity index value can be considered as equivalent. For example, all populations with the same heterozygosity value are equivalent in terms of this index, even if they have radically different alleles frequencies (see Appendix S1 for an example). Moreover, for any given heterozygosity, there will be an “ideal” population in which all alleles are equally frequent. It is therefore possible to define an “effective number of elements” (alleles in this example) as the number of equally frequent elements in an “ideal aggregate” that has the same diversity index value as the “real aggregate.” An example of effective number in an ecological context is the effective number of species introduced by Macarthur (1965) while an equivalent concept in population genetics is the effective number of alleles (Kimura & Crow, 1964).

Note that the concept of effective population size, *N*_*e*_, used in population genetics is analogous to that of Hill numbers but is based on a rather different concept. More precisely, *N*_*e*_ is defined as the number of individuals in an ideal (Wright–Fisher) population that has the same magnitude of random genetic drift as the real population being studied. There are different ways in which we can measure the strength of genetic drift, the most common being change in average inbreeding coefficient, change in allele frequency variance and rate of loss of heterozygosity, and each lead to a different type of effective size. Thus, the ideal and the real populations are equivalent in terms of the rate of loss of genetic diversity and not in terms of equal representation of distinct individuals. Probably the only similarity between *N*_*e*_ and the rationale underlying Hill numbers is in the sense that all the individuals in the ideal population contribute equally (on average) to the gene pool of the next generation.

The application of the above‐stated logic to any of the many different entropy measures used in ecology and population genetics yields a single expression for diversity:

(1) $${}^{q}D\equiv {\left(\sum {}_{i=1}^{S}p_{i}^{q}\right)}^{1/(1-q)},$$

where *S* denotes the number of species or alleles, *p*_*i*_ denotes the relative abundance or frequency of species or allele *i*, and the exponent and superscript *q* is the order of the diversity and indicates the sensitivity of ^*q*^ *D*, the *numbers equivalent* of the diversity measure being used, to common and rare elements (Jost, 2006). The diversity of order zero (*q *= 0) is completely insensitive to species or allele frequencies and is known, respectively, as species or allelic richness depending on whether it is applied to species or allele frequency data. The diversity of order one (*q *= 1) weights the contribution of each species or allele by their frequency without favouring either common or rare species/alleles. Although Equation (1) is not defined for *q* = 1, its limit exists (Jost, 2006):

(2) $${}^{1}D=\exp\left(-\sum_{i=1}^{S}p_{i}\ln p_{i}\right)=\exp\left(H\right)$$

where *H* is the Shannon entropy. All values of *q* greater than unity disproportionally favour the most common species or allele. For example, the Simpson concentration and the Gini–Simpson index, which are, respectively, equivalent to expected homozygosity and expected heterozygosity when applied to allele frequency data, lead to diversities of order 2 and give the same effective number of species or alleles:

(3) $${}^{2}D=1/\left(\sum_{i=1}^{S}p_{i}^{2}\right).$$

It is worth emphasizing that among all these different number equivalents or true diversity measures, the diversity of order 1 is key because of its ability to weigh elements precisely by their frequency without favouring either rare of common elements (Jost, 2006). Therefore, we will use this measure to define our new framework for diversity decomposition.

## 4 WEIGHTED INFORMATION‐BASED DECOMPOSITION FRAMEWORK (*q* = 1)

Our decomposition framework is focused on the information‐based diversity measure (Hill number of order *q* = 1). In what follows, we first describe the framework in terms of abundance (species/genetic) diversities and then we provide an equivalent formulation in terms of phylogenetic diversity. For simplicity, we will use the notation *D* to refer to abundance diversities and PD to refer to phylogenetic diversities both of order *q* = 1. Appendix S2 lists all notation and definitions of the parameters and variables we used.

### 4.1 Formulation in terms of abundance diversity

**Figure 1:**
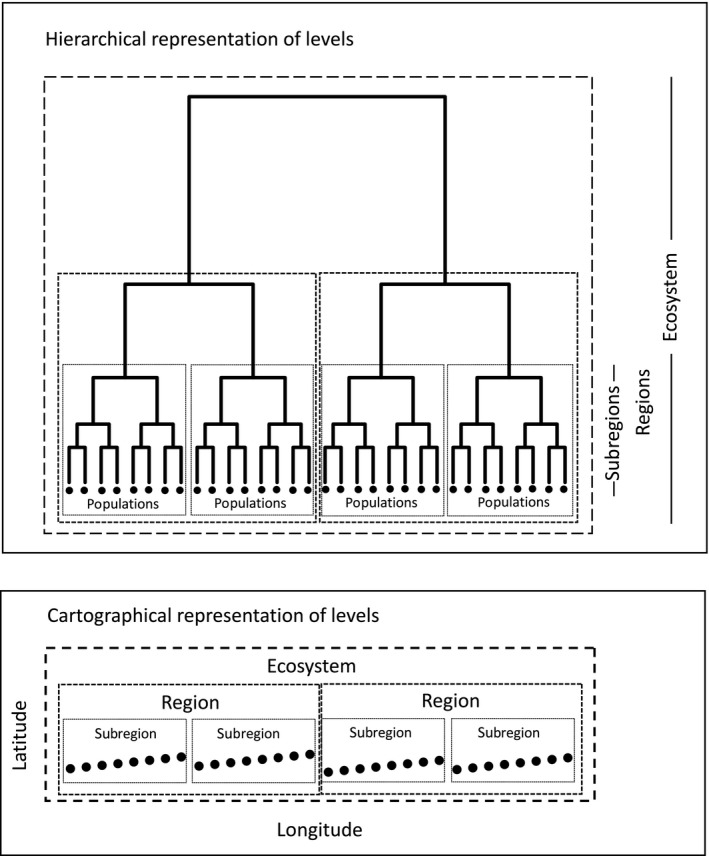
The spatial representation of 32 populations organized into a spatial hierarchy based on three scale levels: subregions (eight populations each), regions (16 populations each) and the ecosystem (all 32 populations). The dendrogram (upper panel—hierarchical representation of levels) represents the spatial relationship (i.e., geographic distance) in which each tip represents a population found in a particular site (lower panel). The cartographic representation (lower panel) represents the spatial distribution of these same populations along a geographic coordinate system

Here, we develop a framework, applicable to both species (abundance, presence–absence, biomass) and genetic data, to estimate alpha, beta and gamma diversities (i.e., diversity components) across different levels of a hierarchical spatial structure. In this section, we consider a very simple example of an ecosystem subdivided into multiple regions, each of which in turn are subdivided into a number of communities when considering species data or a number of populations when considering genetic data. However, our formulation is applicable to any number of levels within a spatially hierarchical partitioning scheme and their associated number of communities and populations at each level (nested scale), such as the example considered in our simulation study below (see Figure 1). Indeed, the framework described here allows decomposing species and genetic information on an equal footing, thus allowing contrasting diversity components across communities and populations. In other words, if genetic and species abundance (or presence–absence) data are available for every population and every species, then genetic and species diversity components can be contrasted within and among spatial scales as well as across different phylogenetic levels. Note that our proposed framework is based on diversities of order *q* = 1, which are less sensitive than diversities of higher order to the fact that genetic information is not available for all individuals in a population but rather based on subsamples of individuals within populations. As such, using *q* = 1 allows one decomposing genetic variation consistently across different spatial subdivision levels that may vary in abundance.

The final objective was to decompose the global (ecosystem) diversity into its regional and community/population‐level components. We do this using the well‐known additive property of Shannon entropy across hierarchical levels (and thus multiplicative partitioning of diversity) (Batty, 1976; Jost, 2007). Table 1 presents the diversities (number equivalents) that need to be estimated at each level of the hierarchy. For each level, there will be one value corresponding to species diversity and another corresponding to allelic (genetic) diversity of a particular species at a given locus (or an average across loci). Figure S1 provides a schematic representation of the calculation of diversities.

**Table 1:** Various diversities in a hierarchically structured system and their decomposition based on diversity measure *D* = ^1^ *D* (Hill number of order *q* = 1 in Equation (2)); for phylogenetic diversity decomposition, replace *D* with PD = ^1^PD (phylogenetic diversity measure of order *q* = 1 in Equation (5)); see Table 3 for all formulas for *D* and PD. The superscripts (1) and (2) denote the hierarchical level of focus

Hierarchical level	Diversity			Decomposition
	Within	Between	Total	
3: Ecosystem	−	−	*D*_γ_	$D_{\gamma }=D_{\alpha }^{\left(1\right)}\;D_{\beta }^{\left(1\right)}D_{\beta }^{\left(2\right)}$
2: Region	$D_{\alpha }^{\left(2\right)}$	$D_{\beta }^{\left(2\right)}=D_{\gamma }^{\left(2\right)}/D_{\alpha }^{\left(2\right)}$	$D_{\gamma }^{\left(2\right)}=D_{\gamma }$	$D_{\gamma }^{}=D_{\alpha }^{\left(2\right)}\;D_{\beta }^{\left(2\right)}$
1: Community or population	$D_{\alpha }^{\left(1\right)}$	$D_{\beta }^{\left(1\right)}=D_{\gamma }^{\left(1\right)}/D_{\alpha }^{\left(1\right)}$	$D_{\gamma }^{\left(1\right)}=D_{\alpha }^{\left(2\right)}$	$D_{\alpha }^{\left(2\right)}=\;D_{\alpha }^{\left(1\right)}D_{\beta }^{\left(1\right)}$

From Table 1, it is apparent that we only need to use Equation (2) to calculate three diversity indices, namely $D_{\alpha }^{\left(1\right)},D_{\alpha }^{\left(2\right)}\;\mathrm{and}\;D_{\gamma }$. These diversity measures are defined in terms of relative abundances of the distinct elements (species or alleles) at the respective levels of the hierarchy. In what follows, we first present the framework as applied to allele count data and then explain how a simple change in the definition of a single parameter allows the application of the same framework to species abundance data. We assume that we are considering a diploid species (but the scheme can be easily generalized for polyploid species) and focus on the diversity of order *q* = 1, which is based on the Shannon entropy (see Equation (1)).

Genetic diversity indices are calculated separately for each locus, so we focus here on a locus with *S* alleles. Additionally, we consider an ecosystem subdivided into *K* regions, each having *J*_*k*_ local populations. Let $N_{i_{n}jk}$ be the number of diploid individuals with *n* (= 0, 1, 2) copies of allele *i* in population *j* and region *k*. Then, the total number of copies of allele *i* in population *j* and region *k* is $N_{\mathrm{ijk}}=\sum_{n=0}^{2}nN_{i_{n}jk}$, and from this, we can derive the total number of alleles in population *j* and region *k* as $N_{+jk}=\sum_{i=1}^{S}N_{\mathrm{ijk}}$, the total number of alleles in region *k* as $N_{++k}=\sum_{j=1}^{J_{k}}N_{+jk}$, and the total number of alleles in the ecosystem as $N_{+++}=\sum_{k=1}^{K}N_{++k}$. All allele frequencies can be derived from these allele counts. For example, the relative frequency of allele *i* in any given population *j* within region *k* is *p*_*i*|*jk*_ = *N*_*ijk*_/*N*_+*jk*_. In the case of region‐ and ecosystem‐level allele frequencies, we pool over populations within regions and over all regions and populations within an ecosystem, respectively. We define the weight for population j and region *k* as *w*_*jk*_ = *N*_+*jk*_/*N*_+++_; the weight for region *k* thus becomes $w_{+k}=\sum_{j=1}^{J_{k}}w_{\mathrm{jk}}=N_{++k}/N_{+++}$. Table 2 describes how allele/species relative frequencies at each level are calculated in terms of these weight functions.

**Table 2:** Calculation of allele/species relative frequencies at the different levels of the hierarchical structure

Hierarchical level	Species/allele relative frequency
Population	$p_{i|jk}=N_{\mathrm{ijk}}/N_{+jk}=N_{\mathrm{ijk}}/\sum_{i=1}^{S}N_{\mathrm{ijk}}\;$
Region	$p_{i|+k}=\;N_{i+k}/N_{++k}=\sum_{j=1}^{J_{k}}\left(w_{\mathrm{jk}}/w_{+k}\right)p_{i|jk}$
Ecosystem	$p_{i|++}=\;N_{i++}/N_{+++}=\sum_{k=1}^{K}\sum_{j=1}^{J_{k}}w_{\mathrm{jk}}p_{i|jk}$

Using these frequencies, we can calculate the genetic diversities at each level of spatial organization. Table 3 presents the formulas for $D_{\alpha }^{\left(1\right)},D_{\alpha }^{\left(2\right)}\;\mathrm{and}\;D_{\gamma }$; all other diversity measures can be derived from them (see Table 1). In the case of the ecosystem diversity, this amounts to simply replacing *p*_*i*_ in Equation (2) by *p*_*i*|++_, the allele frequency at the ecosystem level (see Table 2). To calculate the diversity at the regional level, we first calculate the entropy, $H_{\alpha ,k}^{\left(2\right)}$, for each individual region *k* and then obtain the weighted average over all regions, $H_{\alpha }^{\left(2\right)}$. Finally, we calculate the exponent of the region‐level entropy to obtain $D_{\alpha }^{\left(2\right)}$, the alpha diversity at the regional level. We proceed in a similar fashion to obtain $D_{\alpha }^{\left(1\right)}$, the diversity at the population level but in this case, we need to average over regions and populations within regions.

**Table 3:** Formulas for α,βandγ along with differentiation measures, at each hierarchical level of spatial subdivision for species/allelic diversity and phylogenetic diversity. Here, *D* = ^1^ *D* (Hill number of order *q* = 1 in Equation (2)), PD = ^1^PD (phylogenetic diversity of order *q* = 1 in Equation (5)), *T* denotes the depth of an ultrametric tree. *H* = Shannon entropy (Equation (2)), *I* = phylogenetic entropy (Equation (6))

Hierarchical level	Diversity	Species/allelic diversity	Phylogenetic diversity
Level 3: Ecosystem	gamma	$D_{\gamma }=\exp\left(-\sum_{i=1}^{S}p_{i\left|++\right.}\ln\;p_{i\left|++\right.}\right)$ $\equiv \exp\left(H_{\gamma }\right)$	${\text{PD}}_{\gamma }=T\times \exp\left[\left(-\sum_{i=1}^{B}L_{i}a_{i\left|++\right.}\ln\;a_{i\left|++\right.}\right)/T\right]$ $\equiv T\times \exp\left(I_{\gamma }/T\right)$
Level 2: Region	gamma	$D_{\gamma }^{\left(2\right)}=D_{\gamma }$	${\text{PD}}_{\gamma }^{\left(2\right)}={\text{PD}}_{\gamma }$
	alpha	$D_{\alpha }^{\left(2\right)}=\exp\left(H_{\alpha }^{\left(2\right)}\right)$	${\text{PD}}_{\alpha }^{\left(2\right)}=T\times \exp\left(I_{\alpha }^{\left(2\right)}/T\right)$
		where $H_{\alpha }^{\left(2\right)}=\sum_{k}w_{+k}H_{\alpha ,k}^{\left(2\right)}$	where $I_{\alpha }^{\left(2\right)}=\sum_{k}w_{+k}I_{\alpha ,k}^{\left(2\right)}$
		$H_{\alpha ,k}^{\left(2\right)}=-\sum_{i=1}^{S}p_{i\left|+k\right.}\ln p_{i\left|+k\right.}$	$I_{\alpha ,k}^{\left(2\right)}=-\sum_{i=1}^{B}L_{i}a_{i\left|+k\right.}\ln a_{i\left|+k\right.}$
	beta	$D_{\beta }^{\left(2\right)}=D_{\gamma }^{\left(2\right)}/D_{\alpha }^{\left(2\right)}$	${\text{PD}}_{\beta }^{\left(2\right)}={\text{PD}}_{\gamma }^{\left(2\right)}/{\text{PD}}_{\alpha }^{\left(2\right)}$
Level 1: Population or community	gamma	$D_{\gamma }^{\left(1\right)}=D_{\alpha }^{\left(2\right)}$	${\text{PD}}_{\gamma }^{\left(1\right)}={\text{PD}}_{\alpha }^{\left(2\right)}$
	alpha	$D_{\alpha }^{\left(1\right)}=\exp\left(H_{\alpha }^{\left(1\right)}\right)$	${\text{PD}}_{\alpha }^{\left(1\right)}=T\times \exp\left(I_{\alpha }^{\left(1\right)}/T\right)$
		where $H_{\alpha }^{\left(1\right)}=\sum_{j,k}w_{\mathrm{jk}}H_{\alpha ,jk}^{\left(1\right)}$	where $I_{\alpha }^{\left(1\right)}=\sum_{j,k}w_{\mathrm{jk}}I_{\alpha ,jk}^{\left(1\right)}$
		$H_{\alpha ,jk}^{\left(1\right)}=-\sum_{i=1}^{S}p_{i\left|\mathrm{jk}\right.}\ln p_{i\left|\mathrm{jk}\right.}$	$I_{\alpha ,jk}^{\left(1\right)}=-\sum_{i=1}^{B}L_{i}a_{i\left|\mathrm{jk}\right.}\ln a_{i\left|\mathrm{jk}\right.}$
	beta	$D_{\beta }^{\left(1\right)}=D_{\gamma }^{\left(1\right)}/D_{\alpha }^{\left(1\right)}$	${\text{PD}}_{\beta }^{\left(1\right)}={\text{PD}}_{\gamma }^{\left(1\right)}/{\text{PD}}_{\alpha }^{\left(1\right)}$

Differentiation among aggregates at each level			
Level 2: Among regions		$\Delta_{D}^{\left(2\right)}=\frac{H_{\gamma }-H_{\alpha }^{\left(2\right)}}{-\sum_{k}w_{+k}\ln w_{+k}}$	$\Delta_{\mathrm{PD}}^{\left(2\right)}=\frac{I_{\gamma }-I_{\alpha }^{\left(2\right)}}{-T\sum_{k}w_{+k}\ln w_{+k}}$
Level 1: Population/community within region		$\Delta_{D}^{\left(1\right)}=\frac{H_{\alpha }^{\left(2\right)}-H_{\alpha }^{\left(1\right)}}{-\sum_{j,k}w_{\mathrm{jk}}\ln\left(w_{\mathrm{jk}}/w_{+k}\right)}$	$\Delta_{\mathrm{PD}}^{\left(1\right)}=\frac{I_{\alpha }^{\left(2\right)}-I_{\alpha }^{\left(1\right)}}{-T\sum_{j,k}w_{\mathrm{jk}}\ln\left(w_{\mathrm{jk}}/w_{+k}\right)}$

The calculation of the equivalent diversities based on species count data can be carried out using the exact same procedure described above but in this case, *N*_*ijk*_ represents the number of individuals of species *i* in population *j* and region *k*. All formulas for gamma, alpha and beta, along with the differentiation measures, at each level are given in Table 3. The formulas can be directly generalized to any arbitrary number of levels (see Section 5).

### 4.2 Formulation in terms of phylogenetic diversity

We first present an overview of phylogenetic diversity measures applied to a single nonhierarchical case, henceforth referred to as single aggregate for brevity, and then extend it to consider a hierarchically structured system.

#### 4.2.1 Phylogenetic diversity measures in a single aggregate

To formulate phylogenetic diversity in a single aggregate, we assume that all species or alleles in an aggregate are connected by a rooted ultrametric or nonultrametric phylogenetic tree, with all species/alleles as tip nodes. All phylogenetic diversity measures discussed below are computed from a given fixed tree base or a time reference point that is ancestral to all species/alleles in the aggregate. A convenient time reference point is the age of the root of the phylogenetic tree spanned by all elements. Assume that there are *B* branch segments in the tree, and thus, there are *B* corresponding nodes, *B* ≥ *S*. The set of species/alleles is expanded to include also the internal nodes as well as the terminal nodes representing species/alleles, which will then be the first *S* elements (see Figure S2).

Let *L*_*i*_ denote the length of branch *i* in the tree, *i* = 1, 2, …, *B*. We first expand the set of relative abundances of elements, $\left(p_{1},p_{2},\cdots ,p_{S}\right)$ (see Equation (1)), to a larger set $\left\{a_{i},i=1,2,\cdots ,B\right\}$ by defining *a*_*i*_ as the total relative abundance of the elements descended from the *i*th node/branch, *i* *=* 1, 2, …, *B*. In phylogenetic diversity, an important parameter is the *mean branch length* $\overset{¯}{T}$, the abundance‐weighted mean of the distances from the tree base to each of the terminal branch tips, that is, $\overset{¯}{T}=\sum_{i=1}^{B}L_{i}a_{i}$. For an ultrametric tree, the mean branch length is simply reduced to the *tree depth T*; see Figure 1 in Chao, Chiu, and Jost (2010) for an example. For simplicity, our following formulation of phylogenetic diversity is based on ultrametric trees. The extension to nonultrametric trees is straightforward (via replacing *T* by $\overset{¯}{T}$ in all formulas).

Chao et al. (2010, 2014) generalized Hill numbers to a class of phylogenetic diversity of order *q*, ^*q*^PD, derived as

(4) $${}^{q}\mathrm{PD}={\left\{\sum_{i=1}^{B}L_{i}{\left(\frac{a_{i}}{T}\right)}^{q}\right\}}^{1/(1-q)}.$$

This measure quantifies the effective total branch length during the time interval from *T* years ago to the present. If *q* = 0, then ${}^{0}\mathrm{PD}=\sum_{i=1}^{B}L_{i}$, which is the well‐known *Faith's* PD, the sum of the branch lengths of a phylogenetic tree connecting all species. However, this measure does not consider species abundances. Rao's *quadratic entropy Q* (Rao & Nayak, 1985) is a widely used measure which takes into account both phylogeny and species abundances. This measure is a generalization of the Gini–Simpson index and quantifies the average phylogenetic distance between any two individuals randomly selected from the assemblage. Chao et al. (2010) showed that the ^*q*^PD measure of order *q* = 2 is a simple transformation of quadratic entropy, that is, ${}^{2}\mathrm{PD}=T/\left(1-Q/T\right)$. Again, here we focus on ^*q*^PD measure of order *q* = 1, which can be expressed as a function of the *phylogenetic entropy* (Allen, Kon, & Bar‐Yam, 2009):

(5) $${}^{1}\text{PD}=\lim{}_{q\to 1}{}^{q}\text{PD}=\exp\left[-\sum_{i=1}^{B}L_{i}\frac{a_{i}}{T}\ln\left(\frac{a_{i}}{T}\right)\right]\equiv T\exp\left(I/T\right).$$

Here, *I* denotes the phylogenetic entropy,

(6) $$I=-\sum_{i=1}^{B}L_{i}a_{i}\ln a_{i},$$

which is a generalization of Shannon's entropy that incorporates phylogenetic distances among elements. Note that when there are only tip nodes and all branches have unit length, then we have *T* = 1 and ^*q*^PD reduces to Hill number of order *q* (in Equation (1)).

#### 4.2.2 Phylogenetic diversity decomposition in a multiple‐level hierarchically structured system

The single‐aggregate formulation can be extended to consider a hierarchical spatially structured system. For the sake of simplicity, we consider three levels (ecosystem, region and community/population) as we did for the species/allelic diversity decomposition. Assume that there are *S* elements in the ecosystem. For the rooted phylogenetic tree spanned by all *S* elements in the ecosystem, we define root (or a time reference point), number of nodes/branches *B* and branch length *L*_*i*_ in a similar manner as those in a single aggregate.

For the tip nodes, as in the framework of species and allelic diversity (in Table 2), define, $p_{i\left|\mathrm{jk}\right.}$, $p_{i\left|+k\right.}$ and $p_{i\left|++\right.}$, *i* = 1, 2, …, *S* as the *i*th species or allele relative frequencies at the population, regional and ecosystem level, respectively. To expand these relative frequencies to the branch set, we define $a_{i\left|\mathrm{jk}\right.}$, *i* = 1, 2, …, *B*, as the summed relative abundance of the species/alleles descended from the *i*th node/branch in population *j* and region *k*, with similar definitions for $a_{i\left|+k\right.}$ and $a_{i\left|++\right.}$, *i =* 1, 2, …, *B*; see Figure 1 of Chao et al. (2015) for an illustrative example. The decomposition for phylogenetic diversity is similar to that for Hill numbers presented in Table 1, except that now all measures are replaced by phylogenetic diversity. The corresponding phylogenetic gamma, alpha and beta diversities at each level are given in Table 3, along with the corresponding differentiation measures. Appendix S3 presents all mathematical derivations and discusses the desirable monotonicity and “true dissimilarity” properties that our proposed differentiation measures possess.

## 5 IMPLEMENTATION OF THE FRAMEWORK BY MEANS OF AN R PACKAGE

The framework described above has been implemented in the R function iDIP (information‐based Diversity Partitioning), which is provided as Data S1. We also provide a short introduction with a simple example data set to explain how to obtain numerical results equivalent to those provided in tables 4 and 5 below for the Hawaiian archipelago example data set.

The R function iDIP requires two input matrices:

1. Abundance data: specifying species/alleles (rows) raw or relative abundances for each population/community (columns).
2. Structure matrix: describing the hierarchical structure of spatial subdivision; see a simple example given in Data S1. There is no limit to the number of spatial subdivisions.

The output includes (i) gamma (or total) diversity, alpha and beta diversity for each level, (ii) proportion of total beta information (among aggregates) found at each level and (iii) mean differentiation (dissimilarity) at each level.

We also provide the R function iDIP.phylo, which implements an information‐based decomposition of phylogenetic diversity and, therefore, can take into account the evolutionary history of the species being studied. This function requires the two matrices mentioned above plus a phylogenetic tree in Newick format. For interested users without knowledge of R, we also provide an online version available from https://chao.shinyapps.io/iDIP/. This interactive web application was developed using Shiny (https://shiny.rstudio.com). The webpage contains tabs providing a short introduction describing how to use the tool, along with a detailed User's Guide, which provides proper interpretations of the output through numerical examples.

## 6 SIMULATION STUDY TO SHOW THE CHARACTERISTICS OF THE FRAMEWORK

Here, we describe a simple simulation study to demonstrate the utility and numerical behaviour of the proposed framework. We considered an ecosystem composed of 32 populations divided into four hierarchical levels (ecosystem, region, subregion, population; Figure 1). The number of populations at each level was kept constant across all simulations (i.e., ecosystem with 32 populations, regions with 16 populations each and subregions with eight populations each). Note that here we used a hierarchy with four spatial subdivisions instead of three levels as used in the presentation of the framework. This decision was based on the fact that we wanted to simplify the presentation of calculations (three levels used) and in the simulations (four levels used) we wanted to verify the performance of the framework in a more in‐depth manner.

**Figure 2:**
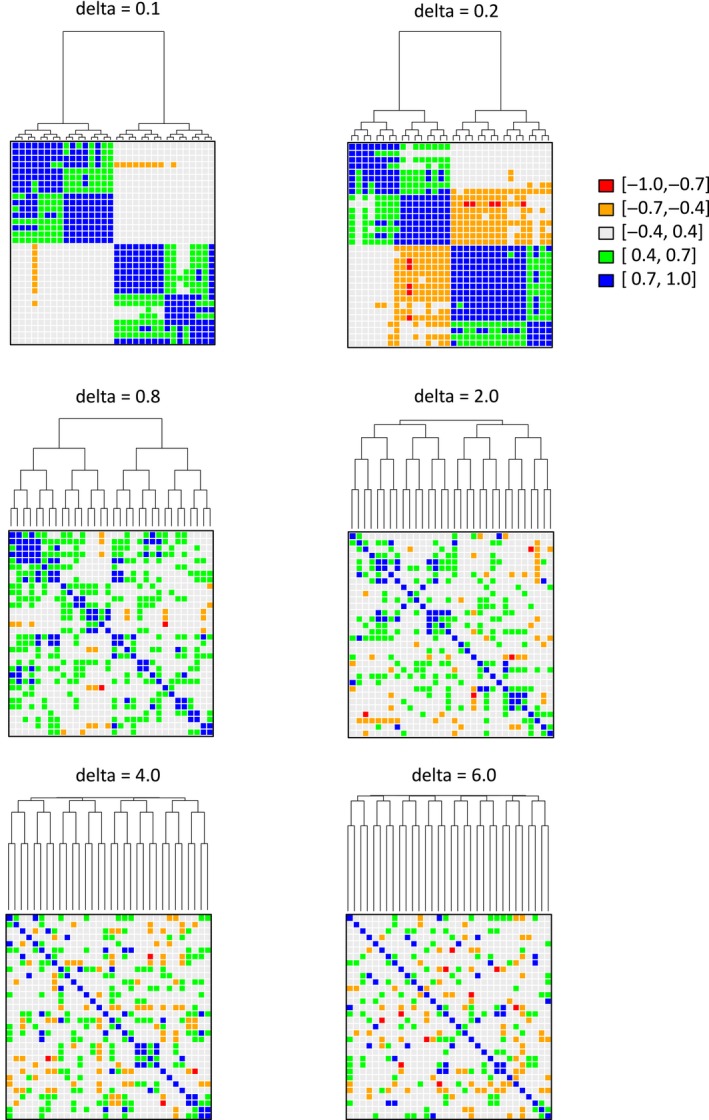
Heatmaps of allele frequency correlations between pairs of populations for different δ values. Delta values control the strength of the spatial genetic structure among populations with low δs having the strongest spatial correlation among populations. Each heatmap represents the outcome of a single simulation and each dot represents the allele frequency correlation between two populations. Thus, the diagonal represents the correlation of a population with itself and is always 1 regardless of the δ value considered in the simulation. Colours indicate range of correlation values. As in Figure 1, the dendrograms represent the spatial relationship (i.e., geographic distance) between populations

We explored six scenarios varying in the degree of genetic structuring, from very strong (Figure 2, top left panel) to very weak (Figure 2, bottom right panel) and, for each, we generated spatially structured genetic data for 10 unlinked bi‐allelic loci using an algorithm loosely based on the genetic model of Coop, Witonsky, Di Rienzo, and Pritchard (2010). More explicitly, to generate correlated allele frequencies across populations for bi‐allelic loci, we draw 10 random vectors of dimension 32 from a multivariate normal distribution with mean zero and a covariance matrix corresponding to the particular genetic structure scenario being considered. To construct the covariance matrix, we first assumed that the covariance between populations decreased with distance so that the off‐diagonal elements (covariances) for closest geographic neighbours were set to 4, for the second nearest neighbours were set to 3 and so on; as such, the main diagonal values (variance) were set to 5. By multiplying the off‐diagonal elements of this variance–covariance matrix by a constant (δ), we manipulated the strength of the spatial genetic structure from strong (δ = 0.1; Figure 2) to weak (δ = 6; Figure 2). Delta values were chosen to demonstrate gradual changes in estimates across diversity components. Using this procedure, we generated a matrix of random normally distributed *N*(0,1) deviates ɛ_*il*_ for each population *i* and locus *l*. The random deviates were transformed into allele frequencies constrained between 0 and 1, using the simple transform:

$$p_{\mathrm{il}}=\left\{\begin{array}{c} 0\;\text{if}\;\varepsilon_{\mathrm{il}}<0\\ \varepsilon_{\mathrm{il}}\;\;\text{if}\;0\leq \varepsilon_{\mathrm{il}}\leq 1,\\ 1\;\text{if}\;\varepsilon_{\mathrm{il}}>1 \end{array}\right.$$

where *p*_*il*_ is the relative frequency of allele A1 at the lth locus in population *i* and, therefore, $q_{\mathrm{il}}=\left(1-p_{\mathrm{il}}\right)$ is the relative frequency of allele A2. Each bi‐allelic locus was analysed separately by our framework, and estimated values of $D_{\gamma },\;D_{\alpha }\;\mathrm{and}\;D_{\beta }$ for each spatial level (see Figure 1) were averaged across the 10 loci.

To simulate a realistic distribution of number of individuals across populations, we generated random values from a log‐normal distribution with mean 0 and log of standard deviation 1; these values were then multiplied by randomly generated deviates from a Poisson distribution with λ = 30, to obtain a wide range of population/community sizes. Rounded values (to mimic abundances of individuals) were then multiplied by *p*_*il*_ and *q*_*il*_ to generate allele abundances. Given that number of individuals was randomly generated across populations, there is no spatial correlation in abundance of individuals across the landscape, which means that the genetic spatial patterns were solely determined by the variance–covariance matrix used to generate correlated allele frequencies across populations. This facilitates interpretation of the simulation results, allowing us to demonstrate that the framework can uncover subtle spatial effects associated with population connectivity (see below).

For each spatial structure, we generated 100 matrices of allele frequencies and each matrix was analysed separately to obtain distributions for $D_{\gamma },\;D_{\alpha },\;D_{\beta }\;\mathrm{and}\;\Delta_{D}$. Figure 2 presents heat maps of the correlation in allele frequencies across populations for one simulated data set under each δ value and shows that our algorithm can generate a wide range of genetic structures comparable to those generated by other more complex simulation protocols (e.g., de Villemereuil, Frichot, Bazin, Francois, & Gaggiotti, 2014).

**Figure 3:**
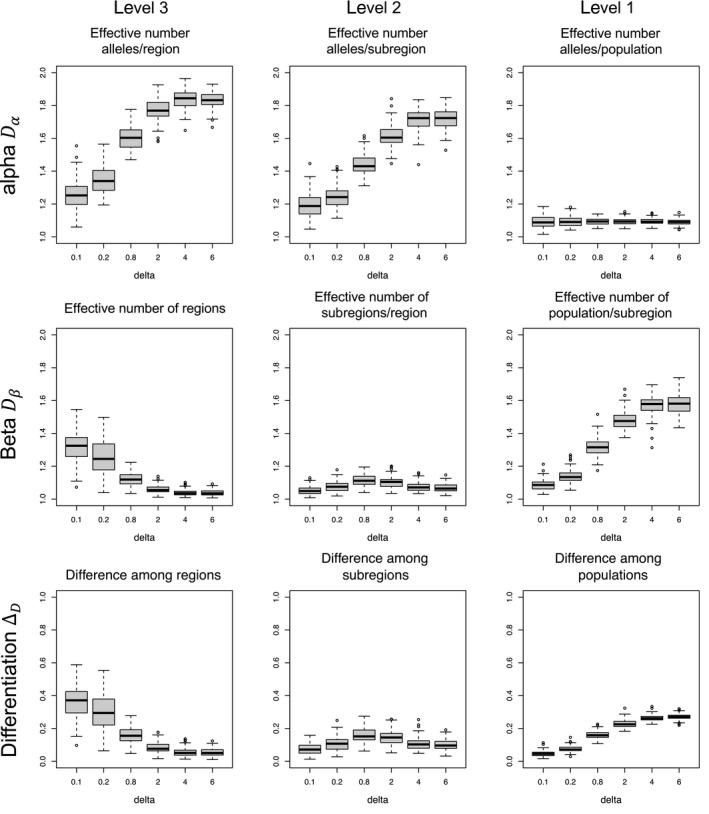
Sampling variation (median, lower and upper quartiles and extreme values) for the three diversity components examined in the simulation study (alpha, beta and differentiation; total diversity gamma is reported in the text only) across 100 simulated populations as a function of the strength (δ values) of the spatial genetic variation among the three spatial levels considered in this study (i.e., populations, subregions and regions)

Figure 3 shows the distribution of $D_{\alpha },\;D_{\beta }\;\mathrm{and}\;\Delta_{D}\;$values for the three levels of geographic variation below the ecosystem level (i.e., *D*_γ_ genetic diversity). The results clearly show that our framework detects differences in genetic diversity across different levels of spatial genetic structure. As expected, the effective number of alleles ($D_{\alpha }$ component, top row) increases per region and subregion as the spatial structure becomes weaker (i.e., from small to large δ values) but remains constant at the population level, as there is no spatial structure at this level (i.e., populations are panmictic) so diversity is independent of δ.

The *D*_β_ component (middle row) quantifies the effective number of aggregates (regions, subregions, populations) at each hierarchical level of spatial subdivision. The larger the number of aggregates at a given level, the more heterogeneous that level is. Thus, it is also a measure of compositional dissimilarity at each level. We use this interpretation to describe the results in a more intuitive manner. As expected, as δ increases dissimilarity between regions (middle left panel) decreases because spatial genetic structure becomes weaker and the compositional dissimilarity among populations within subregions (middle right panel) increases because the strong spatial correlation among populations within subregions breaks down (Figure 3, centre left panel). The compositional dissimilarity between subregions within regions (Figure 3, middle centre panel) first increases and then decreases with increasing δ. This is due to an “edge effect” associated with the marginal status of the subregions at the extremes of the species range (extreme right and left subregions in Figure 2). As δ increases, the composition of the two subregions at the centre of the species range, which belong to different regions, changes more rapidly than that of the two marginal subregions. Thus, the compositional dissimilarity between subregions within regions increases. However, as δ continues to increase, spatial effects disappear and dissimilarity decreases.

The differentiation components $\Delta_{D}$ (bottom row) measures the mean proportion of nonshared alleles in each aggregate and follows the same trends across the strength of the spatial structure (i.e., across δ values) as the compositional dissimilarity *D*_β_. This is expected as we kept the genetic variation equal across regions, subregions and populations. If we had used a nonstationary spatial covariance matrix in which different δ values would be used among populations, subregions and regions, then the beta and differentiation components would follow different trends in relation to the strength in spatial genetic variation.

For the sake of space, we do not show how the total effective number of alleles in the ecosystem (γ diversity) changes as a function of the strength of the spatial genetic structure, but values increase monotonically with δ −*D*_γ_ = 1.6 on average across simulations for δ=0.1 up to $D_{\gamma }=1.9$ for δ = 6. In other words, the effective total number of alleles increases as genetic structure decreases. In terms of an equilibrium island model, this means that migration helps increase total genetic variability. In terms of a fission model without migration, this could be interpreted as a reduced effect of genetic drift as the gene tree approaches a star phylogeny (see Slatkin & Hudson, 1991). Note, however, that these results depend on the total number of populations, which is relatively large in our example; under a scenario where the total number of populations is small, we could obtain a very different result (e.g., migration decreasing total genetic diversity). Our goal here was to present a simple simulation so that users can gain a good understanding of how these components can be used to interpret genetic variation across different spatial scales (here region, subregions and populations). Note that we concentrated on spatial genetic structure among populations as a metric, but we could have used the same simulation protocol to simulate abundance distributions or trait variation among populations across different spatial scales, though the results would follow the same patterns as for the ones we found here. Moreover, for simplicity, we only considered population variation within one species, but multiple species could have been equally considered including a phylogenetic structure among them.

## 7 APPLICATION TO A REAL DATABASE: BIODIVERSITY OF THE HAWAIIAN CORAL REEF ECOSYSTEM

All the above derivations are based on the assumption that we know the population abundances and allele frequencies, which is never true. Instead, estimations are based on allele count samples and species abundance estimations. Usually, these estimations are obtained independently such that the sample size of individuals in a population differs from the sample size of individuals for which we have allele counts. Here, we present an example of the application of our framework to the Hawaiian coral reef ecosystem using fish species density estimates obtained from NOAA cruises (Williams et al., 2015) and microsatellite data for two species, a deep‐water fish *Etelis coruscans* (Andrews et al., 2014) and a shallow‐water fish, *Zebrasoma flavescens* (Eble et al., 2011).

**Figure 4:**
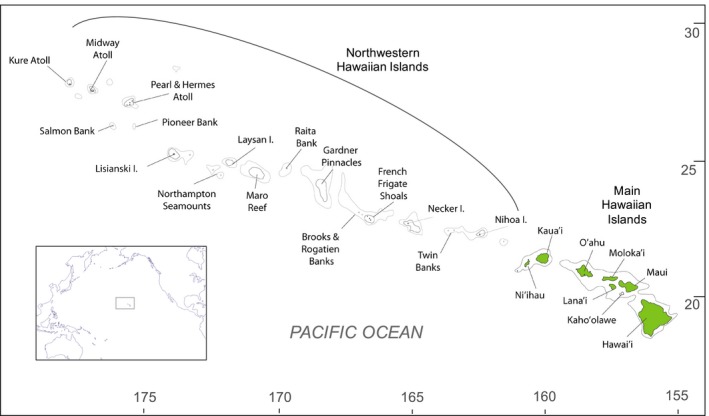
Study domain spanning the Hawaiian Archipelago and Johnston Atoll. Contour lines delineate 1,000 and 2,000 m isobaths. Green indicates large landmass

The Hawaiian archipelago (Figure 4) consists of two regions. The Main Hawaiian Islands (MHI), which are high volcanic islands with many areas subject to heavy anthropogenic perturbations (land‐based pollution, overfishing, habitat destruction and alien species), and the Northwestern Hawaiian Islands (NWHI), which are a string of uninhabited low islands, atolls, shoals and banks that are primarily only affected by global anthropogenic stressors (climate change, ocean acidification and marine debris) (Selkoe et al., 2009). In addition, the northerly location of the NWHI subjects the reefs there to harsher disturbance but higher productivity, and these conditions lead to ecological dominance of endemics over nonendemic fishes (Friedlander, Brown, Jokiel, Smith, & Rodgers, 2003). The Hawaiian archipelago is geographically remote, and its marine fauna is considerably less diverse than that of the tropical West and South Pacific (Randall, 1998). The nearest coral reef ecosystem is 800 km south‐west of the MHI at Johnston Atoll, and is the third region considered in our analysis of the Hawaiian reef ecosystem. Johnston's reef area is comparable in size to that of Maui Island in the MHI, and the fish composition of Johnston is regarded as most closely related to the Hawaiian fish community compared to other Pacific locations (Randall, 1998).

We first present results for species diversity of Hawaiian reef fishes, then for genetic diversity of two exemplar species of the fishes and, finally, address associations between species and genetic diversities. Note that we did not consider phylogenetic diversity in this study because a phylogeny representing the Hawaiian reef fish community is unavailable.

### 7.1 Species diversity

Table 4 presents the decomposition of fish species diversity of order *q* = 1. The effective number of species, *D*_γ_, in the Hawaiian archipelago is 49. In itself, this number is not informative but it would indeed be very useful if we wanted to compare the species diversity of the Hawaiian archipelago with that of other shallow‐water coral reef ecosystem, for example, the Great Barrier Reef. Approximately 10 species equivalents are lost on descending to each lower diversity level in the hierarchy ($\text{Region:}\;D_{\alpha }^{\left(2\right)}=37.77,\;\text{Island:}\;D_{\alpha }^{\left(1\right)}=27.75$). Given that there are eight and nine islands, respectively, in MHI and NWHI, one can interpret this by saying that, on average, each island contains a bit more than one endemic species equivalent. The beta diversity $D_{\beta }^{\left(2\right)}=1.29$ represents the number of region equivalents in the Hawaiian archipelago while $D_{\beta }^{\left(1\right)}=1.361$ is the average number of island equivalents within a region. However, these beta diversities depend on the actual numbers of regions/populations as well as on sizes (weights) of each region/population. Thus, they need to be normalized so as to obtain $\Delta_{D}$ (see bottom section of Table 3) to quantify compositional differentiation. Based on Table 4, the extent of this compositional differentiation in terms of the mean proportion of nonshared species is 0.29 among the three regions (MHI, NWHI and Johnston) and 0.15 among islands within a region. Thus, there is almost twice as much differentiation among regions than among islands within a region.

**Table 4:** Decomposition of fish species diversity of order *q* = 1 and differentiation measures for the Hawaiian coral reef ecosystem

Level	Diversity
3: Hawaiian Archipelago	*D*_γ_ = 48.744
2: Region	$D_{\gamma }^{\left(2\right)}=D_{\gamma },\;D_{\alpha }^{\left(2\right)}=37.773,\;D_{\beta }^{\left(2\right)}=1.290$
1: Island (community)	$D_{\gamma }^{\left(1\right)}=D_{\alpha }^{\left(2\right)},D_{\alpha }^{\left(1\right)}=27.752,\;D_{\beta }^{\left(1\right)}=1.361$

Differentiation among aggregates at each level	
2: Region	$\Delta_{D}^{\left(2\right)}=0.290$
1: Island (community)	$\Delta_{D}^{\left(1\right)}=0.153$

**Figure 5:**
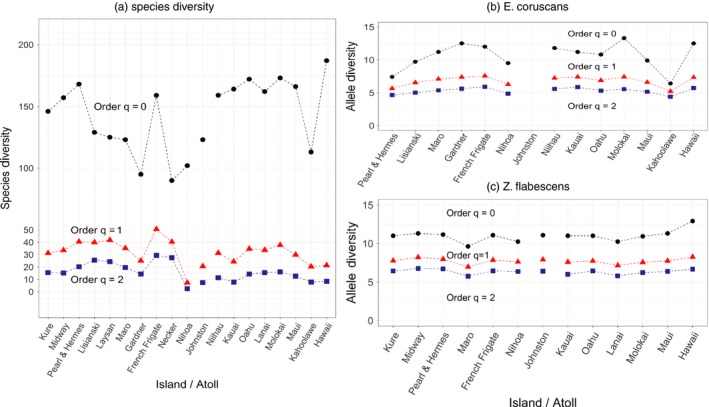
Diversity measures at all sampled islands (communities/populations) expressed in terms of Hill numbers of orders *q* = 0, 1 and 2. (a) Fish species diversity of Hawaiian coral reef communities; (b) genetic diversity for *Etelis coruscans*; (c) genetic diversity for *Zebrasoma flavescens*

We can gain more insight about dominance and other assemblage characteristics by comparing diversity measures of different orders (*q* = 0, 1, 2) at the individual island level (Figure 5a). This is so because the contribution of rare alleles/species to diversity decreases as *q* increases. Species richness (diversity of order *q* = 0) is much larger than those of order *q* = 1, 2, which indicates that all islands contain several rare species. Conversely, diversities of order *q* = 1 and 2 for Nihoa (and to a lesser extent Necker) are very close, indicating that the local community is dominated by few species. Indeed, in Nihoa, the relative density of one species, *Chromis vanderbilti*, is 55.1%.

**Figure 6:**
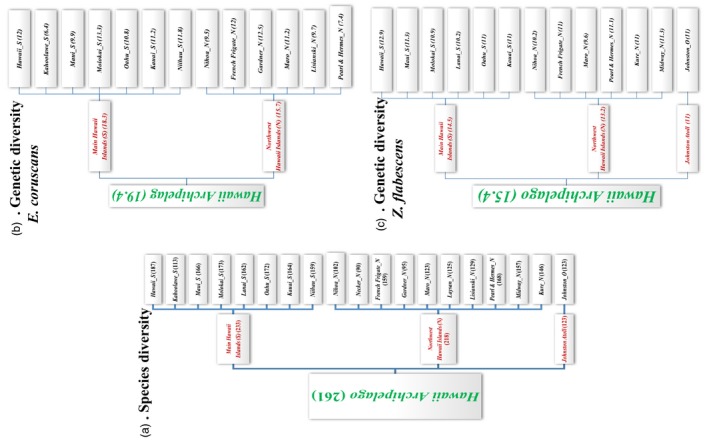
Diagrammatic representation of the hierarchical structure underlying the Hawaiian coral reef database showing observed species/allelic richness (in parentheses) for the Hawaiian coral fish species. (a) Species richness; (b) allelic richness for *Etelis coruscans*; (c) allelic richness for *Zebrasoma flavescens*

Finally, species diversity is larger in MHI than in NWHI (Figure 6a). Possible explanations for this include better sampling effort in the MHI and higher average physical complexity of the reef habitat in the MHI (Friedlander et al., 2003). Reef complexity and environmental conditions may also lead to more evenness in the MHI. For instance, the local adaptation of NWHI endemics allows them to numerically dominate the fish community, and this skews the species abundance distribution to the left, whereas in the MHI, the more typical tropical conditions may lead to competitive equivalence of many species. Although MHI have greater human disturbance than NWHI, each island has some areas of low human impact and this may prevent human impact from influencing island‐level species diversity.

### 7.2 Genetic Diversity

Tables 5 and 6 present the decomposition of genetic diversity for *Etelis coruscans* and *Zebrasoma flavescens*, respectively. They both maintain similar amounts of genetic diversity at the ecosystem level, about eight allele equivalents, and in both cases, genetic diversity at the regional level is only slightly higher than that maintained at the island level (less than one allele equivalent higher), a pattern that contrast with what is observed for species diversity (see above). Finally, both species exhibit similar patterns of genetic structuring, with differentiation between regions being less than half that observed among populations within regions. Note that this pattern contrasts with that observed for species diversity, in which differentiation was greater between regions than between islands within regions. Note also that, despite the similarities in the partitioning of genetic diversity across spatial scales, genetic differentiation is much stronger in *E. coruscans* than *Z. flavescens*, a difference that may be explained by the fact that the deep‐water habitat occupied by the former may have lower water movement than the shallow waters inhabited by the latter and, therefore, may lead to large differences in larval dispersal potential between the two species.

**Table 5:** Decomposition of genetic diversity of order *q* = 1 and differentiation measures for Etelis coruscans. Values correspond to average over 10 loci

Level	Diversity
3: Hawaiian Archipelago	*D*_γ_ = 8.249
2: Region	$D_{\gamma }^{\left(2\right)}=D_{\gamma },\;D_{\alpha }^{\left(2\right)}=8.083,\;D_{\beta }^{\left(2\right)}=1.016$
1: Island (population)	$D_{\gamma }^{\left(1\right)}=D_{\alpha }^{\left(2\right)},D_{\alpha }^{\left(1\right)}=7.077,\;D_{\beta }^{\left(1\right)}=1.117$

Differentiation among aggregates at each level	
2: Region	$\Delta_{D}^{\left(2\right)}=0.023$
1: Island (community)	$\Delta_{D}^{\left(1\right)}=0.062$

**Table 6:** Decomposition of genetic diversity of order *q* = 1 and differentiation measures for Zebrasoma flavescens. Values correspond to averages over 13 loci

Level	Diversity
3: Hawaiian Archipelago	*D*_γ_ = 8.404
2: Region	$D_{\gamma }^{\left(2\right)}=D_{\gamma },\;D_{\alpha }^{\left(2\right)}=8.290,\;D_{\beta }^{\left(2\right)}=1.012$
1: Island (community)	$D_{\gamma }^{\left(1\right)}=D_{\alpha }^{\left(2\right)},D_{\alpha }^{\left(1\right)}=7.690,\;D_{\beta }^{\left(1\right)}=1.065$

Differentiation among aggregates at each level	
2: Region	$\Delta_{D}^{\left(2\right)}=0.014$
1: Island (community)	$\Delta_{D}^{\left(1\right)}=0.033$

Overall, allelic diversity of all orders (*q* = 0, 1, 2) is much less spatially variable than species diversity (Figure 5). This is particularly true for *Z. flavescens* (Figure 5c), whose high larval dispersal potential may help maintain similar genetic diversity levels (and low genetic differentiation) across populations.

As it was the case for species diversity, genetic diversity in MHI is somewhat higher than that observed in NWHI despite its higher level of anthropogenic perturbations (Figure 6b,c).

## 8 DISCUSSION

Biodiversity is an inherently hierarchical concept covering several levels of organization and spatial scales. However, until now, we did not have a framework for measuring all spatial components of biodiversity applicable to both genetic and species diversities. Here, we use an information‐based measure (Hill number of order *q* = 1) to decompose global genetic and species diversity into their various regional‐ and community/population‐level components. The framework is applicable to hierarchical spatially structured scenarios with any number of levels (ecosystem, region, subregion, …, community/population). We also developed a similar framework for the decomposition of phylogenetic diversity across multiple‐level hierarchically structured systems. To illustrate the usefulness of our framework, we used both simulated data with known diversity structure and a real data set stressing the importance of the decomposition for various applications including biological conservation. In what follows, we first discuss several aspects of our formulation in terms of species and genetic diversity and then briefly address the formulation in terms of phylogenetic diversity.

Hill numbers are parameterized by order *q*, which determines the sensitivity of the diversity measure to common and rare elements (alleles or species). Our framework is based on a Hill number of order *q* = 1, which weights all elements in proportion to their frequency and leads to diversity measures based on Shannon's entropy. This is a fundamentally important property from a population genetics point of view because it contrasts with measures based on heterozygosity, which are of order *q* = 2 and, therefore, give a disproportionate weight to common alleles. Indeed, it is well known that heterozygosity and related measures are insensitive to changes in the allele frequencies of rare alleles (e.g., Allendorf, Luikart, & Aitken, 2012) so they perform poorly when used on their own to detect important demographic changes in the evolutionary history of populations and species (e.g., bottlenecks). That said, it is still very useful to characterize diversity of local populations and communities using Hill numbers of order *q* = 0, 1, 2 to obtain a comprehensive description of biodiversity at this scale. For example, a diversity of order *q* = 0 much larger than those of order *q* = 1, 2 indicates that populations/communities contain several rare alleles/species so that alleles/species relative frequencies are highly uneven. Also, very similar diversities of order *q* = 1, 2 indicate that the population/community is dominated by few alleles/species. We exemplify this use with the analysis of the Hawaiian archipelago data set (Figure 5). A continuous diversity profile which depicts Hill number with respect to the order *q* ≥ 0 contains all information about alleles/species abundance distributions.

As proved by Chao et al. (2015, appendix S6) and stated in Appendix S3, information‐based differentiation measures, such as those we propose here (Table 3), possess two essential monotonicity properties that heterozygosity‐based differentiation measures lack: (i) they never decrease when a new unshared allele is added to a population and (ii) they never decrease when some copies of a shared allele are replaced by copies of an unshared allele. Chao et al. (2015) provide examples showing that the commonly used differentiation measures of order *q *= 2, such as *G*_ST_ and Jost's *D*, do not possess any of these two properties.

Other uniform analyses of diversity based on Hill numbers focus on a two‐level hierarchy (community and meta‐community) and provide measures that could be applied to species abundance and allele count data, as well as species distance matrices and functional data (e.g., Chiu & Chao, 2014; Kosman, 2014; Scheiner, Kosman, Presley, & Willig, 2017a,2017b). However, ours is the only one that presents a framework that can be applied to hierarchical systems with an arbitrary number of levels and can be used to derive proper differentiation measures in the range [0, 1] at each level with desirable monotonicity and “true dissimilarity” properties (Appendix S3). Therefore, our proposed beta diversity of order *q* = 1 at each level is always interpretable and realistic, and our differentiation measures can be compared among hierarchical levels and across different studies. Nevertheless, other existing frameworks based on Hill numbers may be extended to make them applicable to more complex hierarchical systems by focusing on diversities of order *q* = 1.

Recently, Karlin and Smouse (2017; Appendix S1) derived information‐based differentiation measures to describe the genetic structure of a hierarchically structured population. Their measures are also based on Shannon entropy/diversity, but they differ in two important aspects from our measures. Firstly, our proposed differentiation measures possess the “true dissimilarity” property (Chao et al., 2014; Wolda, 1981) whereas theirs do not. In ecology, the property of “true dissimilarity” can be enunciated as follows: If *N* communities each have *S* equally common species, with exactly *A* species shared by all of them, and with the remaining species in each community not shared with any other community, then any sensible differentiation measure must give 1 − *A*/*S*, the true proportion of nonshared species in a community. Karlin and Smouse's (2017) measures are useful in quantifying other aspects of differentiation among aggregates, but do not measure “true dissimilarity.” Consider a simple example: populations I and II each has 10 equally frequent alleles, with 4 shared, then intuitively any differentiation measure must yield 60%. However, Karlin and Smouse's measure in this simple case yields 31.96%; on the other hand, ours gives the true nonshared proportion of 60%. The second important difference is that, when there are only two levels, our information‐based differentiation measure reduces to the normalized mutual information (Shannon differentiation), whereas theirs does not. Sherwin (2010) indicated that the mutual information is linearly related to the chi‐square statistic for testing allelic differentiation between populations. Thus, our measures can be linked to the widely used chi‐square statistic, whereas theirs cannot.

In this paper, all diversity measures (alpha, beta and gamma diversities) and differentiation measures are derived conditional on knowing true species richness and species abundances. In practice, species richness and abundances are unknown; all measures need to be estimated from sampling data. When there are undetected species or alleles in a sample, the undersampling bias for the measures of order *q* = 2 is limited because they are focused on the dominant species or alleles, which would be surely observed in any sample. For information‐based measures, it is well known that the observed entropy/diversity (i.e., by substituting species sample proportions into the entropy/diversity formulas) exhibits negative bias to some extent. Nevertheless, the undersampling bias can be largely reduced by novel statistical methods proposed by Chao and Jost (2015). In our real data analysis, statistical estimation was not applied because the patterns based on the observed and estimated values are generally consistent. When communities or populations are severely undersampled, statistical estimation should be applied to reduce undersampling bias. A more thorough discussion of the statistical properties of our measures will be presented in a separate study. Here, our objective was to introduce the information‐based framework and explain how it can be applied to real data.

Our simulation study clearly shows that the diversity measures derived from our framework can accurately describe complex hierarchical structures. For example, our beta diversity $D_{\beta }$ and differentiation $\Delta_{D}$ measures can uncover the increase in differentiation between marginal and well‐connected subregions within a region as spatial correlation across populations (controlled by the parameter δ in our simulations) diminishes (Figure 3). Indeed, the strength of the hierarchical structure varies in a complex way with δ. Structuring within regions declines steadily as δ increases but structuring between subregions within a region first increases and then decreases as δ increases (see Figure 2). Nevertheless, for very large values of δ, hierarchical structuring disappears completely across all levels generating spatial genetic patterns similar to those observed for the island model. A more detailed explanation of the mechanisms involved is presented in the results section.

The application of our framework to the Hawaiian coral reef data allows us to demonstrate the intuitive and straightforward interpretation of our diversity measures in terms of effective number of components. The data sets consist of 10 and 13 microsatellite loci covering only a small fraction of the genome of the studied species. However, more extensive data sets consisting of dense SNP arrays are quickly being produced thanks to the use of next‐generation sequencing techniques. Although SNPs are bi‐allelic, they can be generated in very large numbers covering the whole genome of a species and, therefore, they are more representative of the diversity maintained by a species. Additionally, the simulation study shows that the analysis of bi‐allelic data sets using our framework can uncover complex spatial structures. The R package we provide will greatly facilitate the application of our approach to these new data sets.

Our framework provides a consistent and detailed characterization of biodiversity at all levels of organization, which can then be used to uncover the mechanisms that explain observed spatial and temporal patterns. Although we still have to undertake a very thorough sensitivity analysis of our diversity measures under a wide range of ecological and evolutionary scenarios, the results of our simulation study suggest that diversity measures derived from our framework may be used as summary statistics in the context of Approximate Bayesian Computation methods (Beaumont, Zhang, & Balding, 2002) aimed at making inferences about the ecology and demography of natural populations. For example, our approach provides locus‐specific diversity measures that could be used to implement genome scan approaches aimed at detecting genomic regions subject to selection.

We expect our framework to have important applications in the domain of community genetics. This field is aimed at understanding the interactions between genetic and species diversity (Agrawal, 2003). A frequently used tool to achieve this goal is centred around the study of species–gene diversity correlations (SGDCs). There are now many studies that have assessed the relationship between species and genetic diversity (reviewed by Vellend et al., 2014), but they have led to contradictory results. In some cases, the correlation is positive, in others it is negative, and in yet other cases there is no correlation. These differences may be explained by a multitude of factors, some of which may have a biological underpinning but one possible explanation is that the measurement of genetic and species diversity is inconsistent across studies and even within studies. For example, some studies have correlated species richness, a measure that does not consider abundance, with gene diversity or heterozygosity, which are based on the frequency of genetic variants and give more weight to common than rare variants. In other cases, studies used consistent measures but these were not accurate descriptors of diversity. For example, species and allelic richness are consistent measures but they ignore an important aspect of diversity, namely the abundance of species and allelic variants. Our new framework provides “true diversity” measures that are consistent across levels of organization and, therefore, they should help improve our understanding of the interactions between genetic and species diversities. In this sense, it provides a more nuanced assessment of the association between spatial structuring of species and genetic diversity. For example, a first but somewhat limited application of our framework to the Hawaiian archipelago data set uncovers a discrepancy between species and genetic diversity spatial patterns. The difference in species diversity between regional and island levels is much larger (26%) than the difference in genetic diversity between these two levels (12.44% for *E. coruscans* and 7% for *Z. flavescens*). Moreover, in the case of species diversity, differentiation among regions is much stronger than among populations within regions, but we observed the exact opposite pattern in the case of genetic diversity, genetic differentiation is weaker among regions than among islands within regions. This clearly indicates that species and genetic diversity spatial patterns are driven by different processes.

In our hierarchical framework and analysis based on Hill number of order *q* = 1, all species (or alleles) are considered to be equally distinct from each other such that species (allelic) relatedness is not taken into account: only species abundances are considered. To incorporate evolutionary information among species, we have also extended Chao et al. (2010)'s phylogenetic diversity of order *q* = 1 to measure hierarchical diversity structure from genes to ecosystems (Table 3, last column). Chao et al. (2010)'s measure of order *q* = 1 reduces to a simple transformation of the phylogenetic entropy, which is a generalization of Shannon's entropy that incorporates phylogenetic distances among species (Allen et al., 2009). We have also derived the corresponding differentiation measures at each level of the hierarchy (bottom section of Table 3). Note that a phylogenetic tree encapsulates all the information about relationships among all species and individuals or a subset of them. Our proposed dendrogram‐based phylogenetic diversity measures make use of all such relatedness information.

There are two other important types of diversity that we do not directly address in our formulation. These are trait‐based functional diversity and molecular diversity based on DNA sequence data. In both of these cases, data at the population or species level is transformed into pairwise distance matrices. However, information contained in a distance matrix differs from that provided by a phylogenetic tree. Petchey and Gaston (2002) applied a clustering algorithm to the species pairwise distance matrix to construct a functional dendrogram and then obtain functional diversity measures. An unavoidable issue in their approach is how to select a distance metric and a clustering algorithm to construct the dendrogram; both distance metrics and clustering algorithm may lead to a loss or distortion of species and DNA sequence pairwise distance information. Indeed, Mouchet et al. (2008) demonstrated that the results obtained using this approach are highly dependent on the clustering method being used. Moreover, Maire, Grenouillet, Brosse, and Villeger (2015) noted that even the best dendrogram is often of very low quality. Thus, we do not necessarily suggest the use of dendrogram‐based approaches focused on trait and DNA sequence data to generate a biodiversity decomposition at different hierarchical scales akin to the one used here for phylogenetic structure. An alternative approach to achieve this goal is to use distance‐based functional diversity measures and several such measures have been proposed (e.g., Chiu & Chao, 2014; Kosman, 2014; Scheiner et al., 2017a,2017b). However, the development of a hierarchical decomposition framework for distance‐based diversity measures that satisfies all monotonicity and “true dissimilarity” properties is mathematically very complex. Nevertheless, we note that we are currently extending our framework to also cover this case.

The application of our framework to molecular data is performed under the assumption of the infinite allele mutation model. Thus, it cannot make use of the information contained in markers such as microsatellites and DNA sequences, for which it is possible to calculate distances between distinct alleles. We also assume that genetic markers are independent (i.e., they are in linkage equilibrium), which implies that we cannot use the information provided by the association of alleles at different loci. This situation is similar to that of functional diversity (see preceding paragraph) and requires the consideration of a distance matrix. More precisely, instead of considering allele frequencies, we need to focus on genotypic distances using measures such as those proposed by Kosman (1996) and Gregorius et al. (Gregorius, Gillet, & Ziehe, 2003). As mentioned before, we are currently extending our approach to distance‐based data so as to obtain a hierarchical framework applicable to both trait‐based functional diversity and DNA sequence‐based molecular diversity.

An essential requirement in biodiversity research is to be able to characterize complex spatial patterns using informative diversity measures applicable to all levels of organization (from genes to ecosystems); the framework we propose fills this knowledge gap and in doing so provides new tools to make inferences about biodiversity processes from observed spatial patterns.

## DATA ARCHIVING STATEMENT

All data used in this manuscript are available in DRYAD (https://doi.org/dx.doi.org/10.5061/dryad.qm288) and BCO‐DMO (http://www.bco-dmo.org/project/552879).
