# Supplementary material for: Diversity from genes to ecosystems: A unifying framework to study variation across biological metrics and scales
Source: Evol Appl. 2018 Feb 20;11(7):1176–93. doi: 10.1111/eva.12593 (PMC6050189; doi:10.1111/eva.12593)
Supplement: Supplementary file 1 [file EVA-11-1176-s001.pdf]

SUPPLEMENTARY INFORMATION

Figure S1: Schematic representation of the calculation of diversities at each level of the hierarchy. The five green rectangles represent local populations, the blue represent regions and the red represent the ecosystem. Shannon entropies, $H_*^{(h)}$ are calculated from allele/species abundances at each level h of the hierarchy and are then transformed into effective numbers using eq. 1.

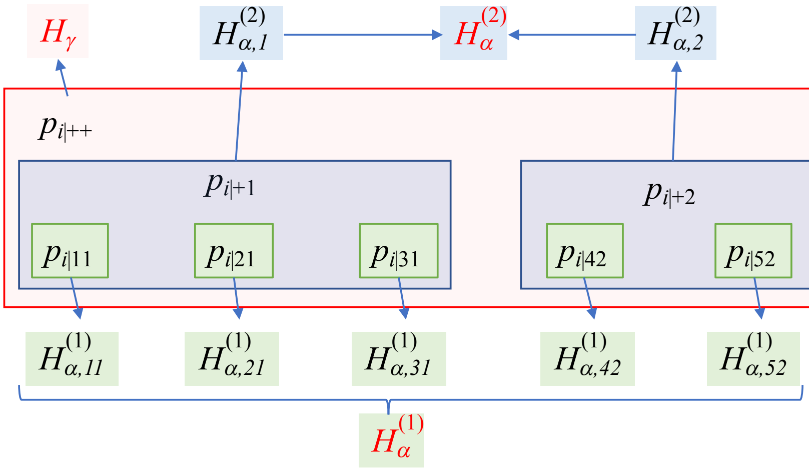

	Within	Between	Total	Decomposition
**3: Ecosystem**	–	–	$D_\gamma = exp(H_\gamma)$	$D_\gamma = D_\alpha^{(1)} D_\beta^{(1)} D_\beta^{(2)}$
**2: Region**	$D_\alpha^{(2)} = exp\left(H_\alpha^{(2)}\right)$	$D_\beta^{(2)} = D_\gamma^{(2)}/D_\alpha^{(2)}$	$D_\gamma^{(2)} = D_\gamma$	$D_\gamma^{(2)} = D_\alpha^{(2)} D_\beta^{(2)}$
**1: Population or Community**	$D_\alpha^{(1)} = exp\left(H_\alpha^{(1)}\right)$	$D_\beta^{(1)} = D_\gamma^{(1)}/D_\alpha^{(1)}$	$D_\gamma^{(1)} = D_\alpha^{(2)}$	$D_\gamma^{(1)} = D_\alpha^{(1)} D_\beta^{(1)}$

Figure S2: Example of an ultrametric tree where the terminal nodes represent species/alleles with associated abundances and the interior nodes representing speciation/coalescent events. In this case the calculation of effective numbers is based on and extended set where the first elements (in the present example 5) correspond to species/alleles abundances and all other abundances correspond to the abundance of the elements descended from the internal nodes. In the present example, the set of abundances for 8 nodes is as follows: $\{a_1, a_2, \cdots, a_5, a_6, a_7, a_8\} = \{p_1, p_2, \cdots, p_5, (p_1 + p_2), (p_1 + p_2 + p_3), (p_4 + p_5)\}$ where p_i is the relative abundance of species *i*.

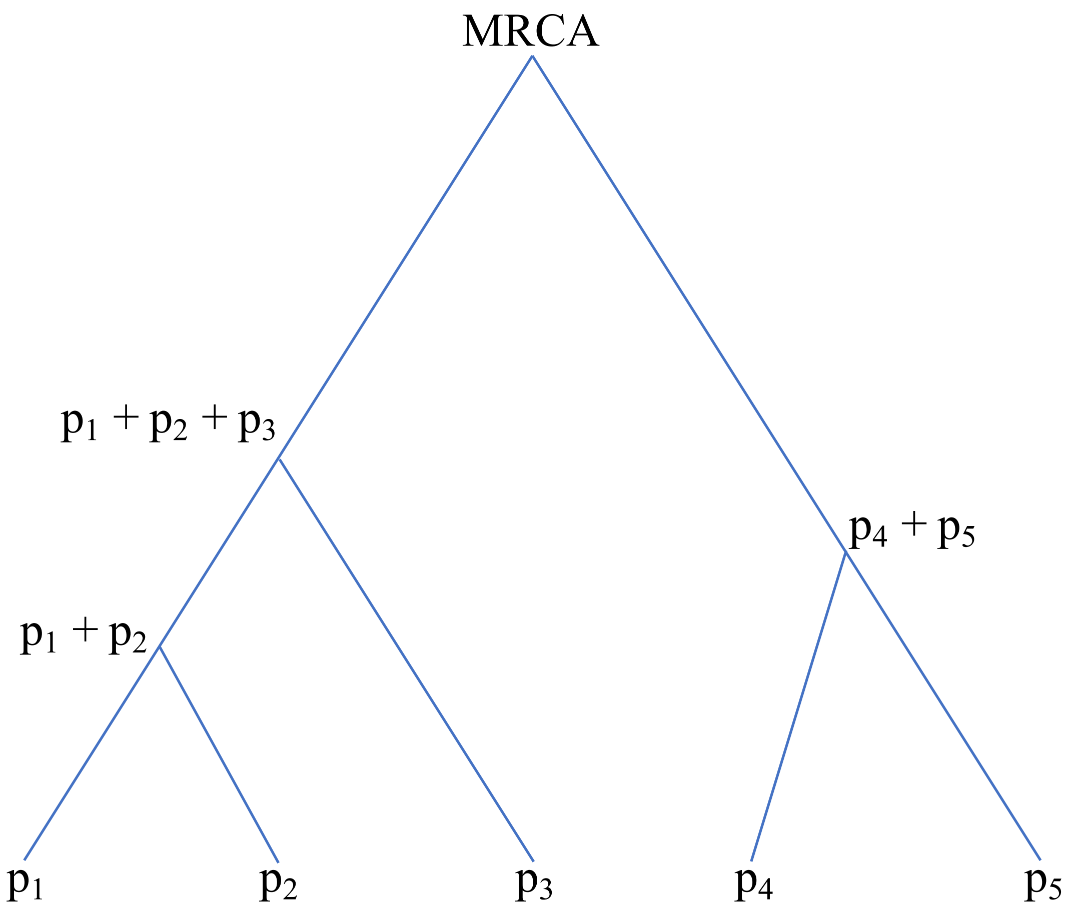

R code for Information-based Diversity Partitioning (iDIP) Under Multi-Level Hierarchical Structures for Species and Phylogenetic Diversities

Species/Allelic Diversity (R Function: iDIP)

Input should include two data matrices (called "Abun" and "Struc" respectively)

(a) Abun: specifying species/alleles (rows) raw or relative frequencies in each population/community (columns); iDIP cannot handle "blank" or "NA" entry. You must replace "blank" or "NA" in your data by 0 or any numerical value. Also, there must be at least one species/allele in a population or a community.

(b) Struc: specifying a hierarchical structure matrix; see a simple example below.

Our R code can be applied to any number of levels. For simplicity, we just use a three-level hierarchical structure to illustrate how to input data. Consider there are two regions (1 and 2) in an ecosystem. In Region 1, there are two populations, and in Region 2, there are three populations. The hierarchical structure is displayed as the following:

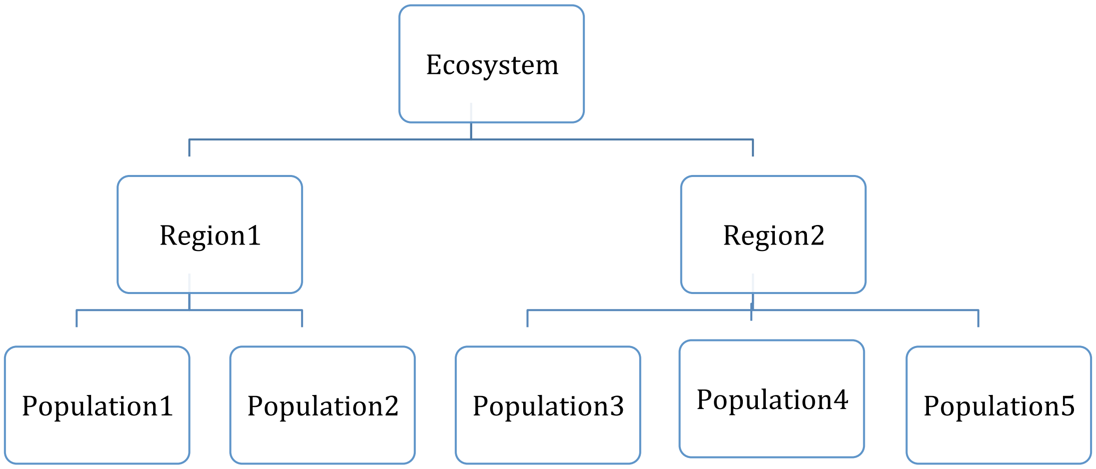

Suppose the raw allele frequencies are given in the following matrix (alleles in rows and populations in columns)

	Pop. 1	Pop. 2	Pop. 3	Pop. 4	Pop. 5
Allele 1	1	16	2	10	15
Allele 2	0	0	0	5	14
Allele 3	7	12	11	1	0
Allele 4	0	5	14	1	21
Allele 5	2	1	0	11	10
Allele 6	0	1	3	2	0

The hierarchical structure matrix for this simple example should be input as a matrix with levels in rows and populations in columns: (Level 1 = population level; Level 2 = region level; Level 3 = ecosystem level.) Hierarchical structure of any number of levels can be expressed in a similar manner.

	Pop. 1	Pop. 2	Pop. 3	Pop. 4	Pop. 5
Level 3	Ecosystem	Ecosystem	Ecosystem	Ecosystem	Ecosystem
Level 2	Region1	Region1	Region2	Region2	Region2
Level 1	Population1	Population2	Population3	Population4	Population5

For the above simple example, input data for R function iDIP (code given below) are shown below.

```
#Input data:
Data=cbind( c(1,0,7,0,2,0),c(16,0,12,5,1,1),c(2,0,11,14,0,3),c(10,5,1,1,11,2),c(15,14,0,21,10,0))
Struc=rbind(rep("Ecosystem",5),c(rep("Region1",2),rep("Region2",3)),paste0("Population",1:5))
#Run R function:
iDIP(Data, Struc)
```

```
#Output is given below
                   [,1]
D_gamma            5.272
D_alpha.2           4.679
D_alpha.1           3.540
D_beta.2           1.127
D_beta.1           1.322
Proportion.2      0.300
Proportion.1      0.700
Differentiation.2 0.204
Differentiation.1 0.310
```

We give simple interpretations for the above output (all the "effective" is in a sense of equally abundant alleles/populations/regions.)

(1a) D_gamma = 5.272 is interpreted as that the effective number of alleles in the ecosystem (total diversity) is 5.272.

(1b) D_alpha.2 = 4.679 is interpreted as that each region contains 4.679 allele equivalents;

D-beta.2 = 1.127 implies that there are 1.13 region equivalents. Thus, 4.679 x 1.127 = 5.272 (=D_gamma).

(1c) D_alpha.1 = 3.540 is interpreted as that each population within a region contains 3.540 allele equivalents;

D_beta.1 =1.322 is interpreted as that there are 1.32 population equivalents per region.

Here 1.322 x 3.540 = 4.679 species per region (= D_alpha.2).

(2) Proportion.2 = 0.30 means that the proportion of total beta information found at the regional level is 30%.

Proportion.1 = 0.70 means that the proportion of total beta information found at the population level is 70%.

(3) Differentiation.2 =0.204 implies that the mean differentiation/dissimilarity among regions is 0.204. This can be interpreted as the following effective sense: the mean proportion of non-shared alleles in a region is around 20.4%.

Differentiation.1 =0.310 implies that the mean differentiation/dissimilarity among

populations within a region is 031, i.e., the mean proportion of non-shared alleles in a population is around 31.0%.

```
##########################################################
##R code for Information-based Diversity Partitioning for Any Number of
Levels:
#input data:
#abun: alleles by population frequency matrix data (or species by
# community frequency matrix data)
#struc: level by population hierarchical structure matrix (or level by community
#hierarchical structure matrix)

#output:
#(1) Gamma (or total) diversity; alpha and beta diversity at each level;
#(2) Proportion of total beta information found at each level;
#(3) Differentiation (dissimilarity) for each level: For example, Differentiation.1
#measures the mean dissimilarity among populations (level 1) within a region;
#Differentiation.2 measures the mean dissimilarity among regions (level 2), etc.

# NOTE: iDIP cannot handle "blank" data or "NA" entry. You must replace "blank"
# or "NA" in your data by 0 or any numerical value. Also, there must be at least
# one species/allele in a population or a community.

# Main function iDIP

iDIP=function(abun,struc){
  n=sum(abun);N=ncol(abun);
  ga=rowSums(abun);
  gp=ga[ga>0]/n;
  G=sum(-gp*log(gp))
  S=length(gp);
  H=nrow(struc);
  A=numeric(H-1);W=numeric(H-1);B=numeric(H-1);
```

```
Diff=numeric(H-1);Prop=numeric(H-1);

wi=colSums(abun)/n;
W[H-1]=-sum(wi[wi>0]*log(wi[wi>0]));
pi=sapply(1:N,function(k) abun[,k]/sum(abun[,k]))
Ai=sapply(1:N,function(k) -sum(pi[,k][pi[,k]>0]*log(pi[,k][pi[,k]>0])))
A[H-1]=sum(wi*Ai);
if(H>2){
  for(i in 2:(H-1)){
  I=unique(struc[i,]);NN=length(I);
  ai=matrix(0,ncol=NN,nrow=nrow(abun));
  for(j in 1:NN){
    II=which(struc[i,]==I[j]);
    if(length(II)==1) {ai[,j]=abun[,II];
    }else{ai[,j]=rowSums(abun[,II])}
    }
  pi=sapply(1:NN,function(k) ai[,k]/sum(ai[,k]));
  wi=colSums(ai)/sum(ai);
  W[i-1]=-sum(wi*log(wi))
  Ai=sapply(1:NN,function(k) -sum(pi[,k][pi[,k]>0]*log(pi[,k][pi[,k]>0])))
  A[i-1]=sum(wi*Ai);
  }
  }
 total=G-A[H-1];
 Diff[1]=(G-A[1])/W[1];
 Prop[1]=(G-A[1])/total;
 B[1]=exp(G)/exp(A[1]);
 if(H>2){
 for(i in 2:(H-1)){
   Diff[i]=(A[i-1]-A[i])/(W[i]-W[i-1]);
   Prop[i]=(A[i-1]-A[i])/total;
   B[i]=exp(A[i-1])/exp(A[i]);
 }}
 Gamma=exp(G);Alpha=exp(A);Diff=Diff;Prop=Prop;
```

```
    out=matrix(c(Gamma,Alpha,B,Prop,Diff),ncol=1)
    rownames(out) <- c(paste0("D_gamma"),
                       paste0("D_alpha.", (H-1):1),
                       paste0("D_beta.", (H-1):1),
                       paste0("Proportion.", (H-1):1),
                       paste0("Differentiation.",(H-1):1)
                       )
    return(out)
}
############################################################
```

Phylogenetic Diversity (R function: iDIP.phylo)

In addition to the two data matrices (called "Abun", "Struc" respectively) as described in the species diversity, we also need to input a phylogenetic "Tree" in Newick tree format.)

(a) Abun: specifying species/alleles (rows) raw or relative frequencies in each population/community (columns).
NOTE: species names in the "Abun" matrix should be exactly the same as those in the uploaded Newick tree format. iDIP cannot handle "blank" or "NA" entry. You must replace "blank" or "NA" in your data by 0 or any numerical value. Also, there must be at least one species/allele in a population or a community.

(b) Struc: specifying hierarchical structure matrix; see the simple example given above for the species diversity.

(c) Tree: a phylogenetic tree spanned by all species considered in the study.

Here we use the same hierarchical structure and allele abundances data as in the species diversity for illustration. A simulated phylogenetic tree for 6 species are given below.

```
#Input data:
Data=cbind( c(1,0,7,0,2,0),c(16,0,12,5,1,1),c(2,0,11,14,0,3),c(10,5,1,1,11,2),c(15,14,0,21,10,0))
row.names(Data)= paste0("Allele",1:6);
Struc=rbind(rep("Ecosystem",5),c(rep("Region1",2),rep("Region2",3)),paste0("Community",1:5))
Tree=c("(((Allele1:16.66254448,Allele2:28.86156926):43.70264926,Allele3:59.19367445):43.49065302,(Allele4:9.67060281,Allele5:49.65919121,Allele6:15.361314):54.92297125);")

#Run R function:
iDIP.phylo(Data, Struc, Tree)

#Output is given below:
            [,1]
```

```
Faith's PD 321.525
mean_T      94.169
PD_gamma   274.388
PD_alpha.2 255.194
PD_alpha.1 223.231
PD_beta.2    1.075
PD_beta.1    1.143
PD_prop.2    0.351
PD_prop.1    0.649
PD_diff.2    0.124
PD_diff.1    0.149
```

The “effective” in the following interpretation is in the sense of equally abundant and equally divergent lineages/communities/regions.

(1) The total branch length (Faith’s PD) in the phylogenetic tree is 321.525.

(2) The weighted (by species abundance) mean of the distances from root node to each of the tips is 94.169.

(3a) PD_gamma = 274.388 is interpreted as that the effective total branch length in the ecosystem (total phylogenetic diversity) is 274.388.

(3b) PD_alpha.2 = 255.194 is interpreted as that the effective total branch length per region is 255.194.

PD-beta.2 = 1.075 means that there are 1.08 region equivalents. Thus, 255.194 x 1.075 = 274.388 (=PD_gamma).

(3c) PD_alpha.1 =223.231 is interpreted as that the effective total branch length per population within each region is 223.231.

PD_beta.1 =1.143 implies that there are 1.14 population equivalents per region. Here 223.231 x 1.143 = 255.194 (= PD_alpha.2).

(4) PD_prop.2 = 0.351 means that the proportion of total phylogenetic beta information found in the regional level is 35.1%.

PD_prop.1 = 0.649 means that the proportion of total phylogenetic beta information found in the community level is 64.9%.

(5) PD_diff.2 =0.124 implies that the mean phylogenetic differentiation among regions is 0.124. This can be interpreted as the following effective sense: the mean proportion of non-shared lineages in a region is around 12.5%.

PD_diff.1 =0.149 implies that the mean phylogenetic differentiation among communities within a region is 0.149, i.e., the mean proportion of non-shared lineages in a community is around 14.9%.

```
########################################################
##R code for Phylogenetic-Information-Based Decomposition for Any
##Number of Levels:
#input data:
#abun: alleles by population frequency matrix data (or species by community
# frequency matrix data)
#struc: level by population hierarchical structure matrix (or level by community
#hierarchical structure matrix
#tree: a Newick-format phylogenetic tree spanned by all focal species considered
# in a study

# NOTE: iDIP cannot handle "blank" or "NA" entry. You must replace blank
# or "NA" in your data by 0 or any numerical value. Also, there must be at least
# one species/allele in a population or a community.

#output:
#(1) Faith's PD: the total sum of branch lengths of a phylogenetic tree
#(2) mean T: weighted (by species abundance) mean of the distances from root
# node to each of the tips in a phylogenetic tree. For an ultrametric tree,
# mean T = tree depth.
#(3) Gamma (or total) phylogenetic diversity (PD) of order 1; alpha and beta PD
# for each level;
#(4) PD_Prop.1 and PD_prop.2 measure the proportions of total phylogenetic
#beta information found in Level 1 and Level 2, respectively;
#(5) Phylogenetic differentiation (dissimilarity) for each level: For example,
#PD_diff.1 measures (level-1) the mean phylogenetic dissimilarity among
#communities (Level 1) within a region (Level 2); PD_diff.2 measures the mean
#phylogenetic dissimilarity among regions (Level 2), etc.

#Three packages "ade4", "ape" and "phytools" must be installed first.
```

```
install.packages("ade4")
library(ade4)
install.packages("ape")
library(ape)
install.packages("phytools")
library(phytools)
```

Main function iDIP.phylo

```
iDIP.phylo=function(abun,struc,tree){
  phyloData <- newick2phylog(tree)
  Temp <- as.matrix(abun[names(phyloData$leaves), ])

  nodenames=c(names(phyloData$leaves),names(phyloData$nodes));

M=matrix(0,nrow=length(phyloData$leaves),ncol=length(nodenames),dimname
s=list(names(phyloData$leaves),nodenames))

  for(i in
1:length(phyloData$leaves)){M[i,][unlist(phyloData$paths[i])]=rep(1,length(unl
ist(phyloData$paths[i])))}


pA=matrix(0,ncol=ncol(abun),nrow=length(nodenames),dimnames=list(nodena
mes,colnames(abun)))
  for(i in 1:ncol(abun)){pA[,i]=Temp[,i]%*%M;}
  pB=c(phyloData$leaves,phyloData$nodes)

  n=sum(abun);N=ncol(abun);
  ga=rowSums(pA);
  gp=ga/n;TT=sum(gp*pB);
  G=sum(-pB[gp>0]*gp[gp>0]/TT*log(gp[gp>0]/TT))

  PD=sum(pB[gp>0]);
```

```
   H=nrow(struc);
   A=numeric(H-1);W=numeric(H-1);B=numeric(H-1);
   Diff=numeric(H-1);Prop=numeric(H-1);

   wi=colSums(abun)/n;
   W[H-1]=-sum(wi[wi>0]*log(wi[wi>0]));
   pi=sapply(1:N,function(k) pA[,k]/sum(abun[,k]))
   Ai=sapply(1:N,function(k)
-sum(pB[pi[,k]>0]*pi[,k][pi[,k]>0]/TT*log(pi[,k][pi[,k]>0]/TT)))
   A[H-1]=sum(wi*Ai);

   if(H>2){
      for(i in 2:(H-1)){
         I=unique(struc[i,]);NN=length(I);
         pi=matrix(0,ncol=NN,nrow=nrow(pA));ni=numeric(NN);
         for(j in 1:NN){
            II=which(struc[i,]==I[j]);
            if(length(II)==1) {pi[,j]=pA[,II]/sum(abun[,II]);ni[j]=sum(abun[,II]);
            }else{pi[,j]=rowSums(pA[,II])/sum(abun[,II]);ni[j]=sum(abun[,II])}
         }
         #pi=sapply(1:NN,function(k) ai[,k]/sum(ai[,k]));
         wi=ni/sum(ni);
         W[i-1]=-sum(wi*log(wi))
         Ai=sapply(1:NN,function(k)
-sum(pB[pi[,k]>0]*pi[,k][pi[,k]>0]/TT*log(pi[,k][pi[,k]>0]/TT)))
         A[i-1]=sum(wi*Ai);
      }
   }
   total=G-A[H-1];
   Diff[1]=(G-A[1])/W[1];
   Prop[1]=(G-A[1])/total;
   B[1]=exp(G)/exp(A[1]);
   if(H>2){
```

```
    for(i in 2:(H-1)){
      Diff[i]=(A[i-1]-A[i])/(W[i]-W[i-1]);
      Prop[i]=(A[i-1]-A[i])/total;
      B[i]=exp(A[i-1])/exp(A[i]);
    }}

  #Gamma=exp(G)/TT;Alpha=exp(A)/TT;Diff=Diff;Prop=Prop;
  Gamma=exp(G);Alpha=exp(A);Diff=Diff;Prop=Prop;
  out=matrix(c(PD,TT,Gamma,Alpha,B,Prop,Diff),ncol=1)
  #out1=iDIP(abun,struc);
  #out=cbind(out1,out2);

  rownames(out) <- c(paste("Faith's PD"),
                     paste("mean_T"),
                     paste0("PD_gamma"),
                     paste0("PD_alpha.", (H-1):1),
                     paste0("PD_beta.", (H-1):1),
                     paste0("PD_prop.", (H-1):1),
                     paste0("PD_diff.",(H-1):1)
  )
  return(out)
}
```
