# Supplementary material for: Diversity from genes to ecosystems: A unifying framework to study variation across biological metrics and scales
Source: Evol Appl. 2018 Feb 20;11(7):1176–93. doi: 10.1111/eva.12593 (PMC6050189; doi:10.1111/eva.12593)
Supplement: Supplementary file 2 [file EVA-11-1176-s002.pdf]

APPENDIX S1: Effective number of alleles – A simple example

Example of two populations that have radically different allele frequencies but belong to the same equivalence class because they have the same expected heterozygosity. Population 2 with five distinct alleles is equivalent to a population with only two equally abundant alleles. Note also that population 1 has the maximum possible heterozygosity when only two alleles are present. Thus, one can say that it represents an "ideal" population, i.e. a population with the maximum possible diversity given the number of distinct alleles it contains. Therefore, the "effective" number of alleles in population 2 is two.

Allele	Population	
	1	2
A_1	0.5	0.01
A_2	0.5	0.10
A_3	0	0.665
A_4	0	0.215
A_5	0	0.01
H_e	0.5	0.5

APPENDIX S2 – Table of parameters and variables used

Definitions of the various parameters and variables following the order in which they appear in the text.

Symbol	Definition
^{q}D	abundance diversity of order *q*, also referred to as Hill number of order *q*
^{q}PD	phylogenetic diversity of order *q*.
S	total number of distinct elements = species/alleles
H	Shannon entropy
K	number of regions
J_k	number of local populations/communities within region *k*.
w_{jk}	weight given to population/community *j* of region *k*.
w_{+k}	weight given to region *k*.
$D_{\alpha}^{(l)}$	alpha abundance diversity at level *l* of the hierarchy.
$D_{\beta}^{(l)}$	beta abundance diversity at level *l* of the hierarchy.
$D_{\gamma}^{(l)}$	gamma abundance diversity at level *l* of the hierarchy.
l	superscript to denote population/community, subregion, region, …
D_{γ}	gamma abundance diversity at the ecosystem level.
$N_{i_n jk}$	number of individuals with $n = 0,1,2$ copies of allele *i* in population/community *j* of region *k.*
N_{ijk}	total number of copies of allele/species *i* in population/community *j* of region *k*.

$\boldsymbol{N_{+jk}}$	total number of alleles/individuals in population *j* of region *k*.
$\boldsymbol{N_{++k}}$	total number of alleles/individuals in region *k*.
$\boldsymbol{N_{+++}}$	total number of alleles/individuals in the ecosystem.
$\boldsymbol{p_{i\|jk}}$	frequency of allele/species *i* in population *j* of region *k*.
$\boldsymbol{p_{i\|+k}}$	frequency of allele/species *i* in region *k*.
$\boldsymbol{p_{i\|++}}$	frequency of allele/species *i* in the ecosystem.
$\boldsymbol{H_{\alpha,jk}^{(1)}}$	alpha entropy for population *j* of region *k*.
$\boldsymbol{H_{\alpha,k}^{(2)}}$	alpha entropy for region *k*.
$\boldsymbol{H_{\alpha}^{(l)}}$	total alpha entropy at level *l* of the hierarchy.
$\boldsymbol{\Delta_{D}^{(l)}}$	abundance diversity differentiation among aggregates (*l* = populations/communities, regions).
B	number of branch segments in the phylogenetic tree.
$\boldsymbol{L_i}$	length of branch $i = 1,2,3,\cdots,B$.
$\boldsymbol{a_i}$	total relative abundance of elements (alleles/species) descended from the ith node/branch.
$\boldsymbol{\bar{T}}$	mean branch length.
T	depth of an ultrametric tree (= $\bar{T}$)
$\boldsymbol{Q}$	Rao's quadratic entropy.
I	phylogenetic entropy.
$\boldsymbol{a_{i\|jk}}$	total relative abundance of allele/species descended from node *i* in population/community *j* of region *k*.
$\boldsymbol{a_{i\|+k}}$	total relative abundance of allele/species descended from node *i* across all populations/communities in region *k*.
$\boldsymbol{a_{i\|++}}$	total relative abundance of allele/species descended from node *i* across all populations and regions in the ecosystem.
$\boldsymbol{PD_{\alpha}^{(l)}}$	alpha phylogenetic diversity at level *l* of the hierarchy.
$\boldsymbol{PD_{\beta}^{(l)}}$	beta phylogenetic diversity at level *l* of the hierarchy.
$\boldsymbol{PD_{\gamma}^{(l)}}$	gamma phylogenetic diversity at level *l* of the hierarchy.
$\boldsymbol{PD_{\gamma}}$	gamma phylogenetic diversity at the ecosystem level.
$\boldsymbol{I_{\alpha,jk}^{(1)}}$	alpha phylogenetic entropy for population *j* of region *k*.
$\boldsymbol{I_{\alpha,k}^{(2)}}$	alpha phylogenetic entropy for region *k*.
$\boldsymbol{I_{\alpha}^{(l)}}$	total alpha phylogenetic entropy at level *l* of the hierarchy.
$\boldsymbol{\Delta_{PD}^{(l)}}$	phylogenetic diversity differentiation among aggregates (*l* = populations/communities, regions).

APPENDIX S3: Derivation of Differentiation Measures

We derive all differentiation measures in terms of allele frequencies but note that all derivations are valid for species diversity; it suffices to replace the term "allele frequency" with "species frequency". We first present the details for two-level hierarchy and then extend all procedures to three-level hierarchy. Generalization to an arbitrary number of levels is parallel.

Shannon differentiation measure in two-level hierarchy (ecosystem and populations)

Assume that there are *J* populations and *S* alleles in an ecosystem. Denote the relative frequency of allele *i* within population *j* as $p_{i|j}, \sum_{i=1}^{S} p_{i|j} = 1$, for any *j* = 1, 2, …, *J*. For *any* given population weights $(w_1, w_2, \cdots, w_J), \sum_{j=1}^{J} w_j = 1$, the relative frequency of allele *i* in the ecosystem becomes $p_{i|+}, \sum_{j=1}^{J} w_j p_{i|j}$. The gamma and alpha Shannon entropies can be expressed as

$$H_\gamma = -\sum_{i=1}^{S} p_{i|+} \ln p_{i|+} = -\sum_{i=1}^{S}\left(\sum_{j=1}^{J} w_j p_{i|j}\right) \ln\left(\sum_{l=1}^{J} w_j p_{i|l}\right),$$

and

$$H_\alpha = -\sum_{j=1}^{J} w_j \sum_{i=1}^{S} p_{i|j} \ln p_{i|j}.$$

<u>Theorem S3.1.</u> For the gamma and alpha entropies defined above for two-level hierarchy, we have the following inequalities:

$$0 \le H_\gamma - H_\alpha \le -\sum_{j=1}^{J} w_j \ln w_j.$$

When all populations have identical allele relative frequency distributions, we have $H_\gamma - H_\alpha = 0$; when the *J* populations are completely distinct (no shared alleles), we have $H_\gamma - H_\alpha = -\sum_{j=1}^{J} w_j \ln w_j$.

Proof:
Since $f(x) = -x \log x$ is a concave function, it follows from the Jensen inequality that for any allele *i*, we have

$$-\left(\sum_{j=1}^{J} w_j\, p_{i|j}\right) \ln\left(\sum_{j=1}^{J} w_j\, p_{i|j}\right) \ge -\sum_{j=1}^{J} w_j p_{i|j} \ln p_{i|j}.$$

Summing over all alleles, we then obtain

$$-\sum_{i=1}^{S}\left(\sum_{j=1}^{J} w_j\, p_{i|j}\right) \ln\left(\sum_{j=1}^{J} w_j\, p_{i|j}\right) \ge -\sum_{j=1}^{J} w_j \sum_{i=1}^{S} p_{i|j} \ln p_{i|j}.$$

This proves $H_\gamma \ge H_\alpha$. The Jensen inequality become equality if and only if $p_{i|1} = p_{i|2} = \cdots = p_{i|J}$ for any allele *i* = 1, 2, …, *S*, i.e., all *J* populations have identical allele frequency distributions. The maximum value of $H_\gamma - H_\alpha$ is obtained as follows:

$$H_\gamma = -\sum_{i=1}^{S}\left(\sum_{j=1}^{J} w_j p_{i|j} \ln\left(\sum_{l=1}^{J} w_j p_{i|l}\right)\right) \le -\sum_{i=1}^{S}\left(\sum_{j=1}^{J} w_j p_{i|j} \ln w_j p_{i|j}\right)$$

$$= -\sum_{j=1}^{J} w_j \sum_{i=1}^{S} p_{i|j} \ln p_{i|j} - \sum_{j=1}^{J} w_j \sum_{i=1}^{S} p_{i|j} \ln w_j = H_\alpha - \sum_{j=1}^{J} w_j \ln w_j.$$

When all *J* populations are completely distinct (no shared alleles), the above inequality becomes an equality. The proof is thus completed.

From the above theorem, the normalized differentiation measure (Shannon differentiation) is formulated as

$$\Delta_D = \frac{H_\gamma - H_\alpha}{-\sum_{j=1}^{J} w_j \ln w_j}. \qquad \text{(C.1)}$$

This measure takes the minimum value of 0 when all populations have identical allele frequency distributions and it takes the maximum value of 1 when the *J* populations are completely distinct (no shared alleles). Shannon differentiation measure satisfies two monotonicity properties (stated in the first part of the following theorem) that heterozygosity-based measures lack.

<u>Theorem S3.2.</u> Desirable monotonicity and "true dissimilarity" properties for Shannon differentiation

(A) Shannon differentiation (given in Eq. C.1) satisfies the following monotonicity properties that heterozygosity-based measures lack:

(A1) Shannon differentiation always increases when some copies of an allele that is shared between two or more populations are replaced by copies of an unshared allele.

(A2) Shannon differentiation measure is always non-decreasing when a new allele is added to a single population, with any abundance. Here the population weights can be a set of specified weights or relative population sizes.

(B) In addition, Shannon differentiation also satisfies the "true dissimilarity" property: If multiple communities each have *S* equally common species, with exactly *A* species shared by all of them, and with the remaining species in each community not shared with any other community, then Shannon differentiation measure gives 1–*A/S*, the true proportion of non-shared species in a community.

Proof: For Part (A), see Appendix S6 in Chao, Jost, et al. (2015) for proof details and counter-examples; for Part (B), see Chao and Chiu (2016).

Shannon differentiation measures in three-level hierarchy

Consider the three-level hierarchy (ecosystem-region-population) in Table 1 of the main text. From Table 3 of the main text, Shannon gamma and alpha entropies are expressed as:

$$H_\gamma = -\sum_{i=1}^{S} p_{i|++} \ln p_{i|++} \ , \ H_\alpha^{(2)} = -\sum_{k=1}^{K} w_{+k} \sum_{i=1}^{S} p_{i|+k} \ln p_{i|+k},$$
$$H_\alpha^{(1)} = -\sum_{k=1}^{K} \sum_{j=1}^{J_k} w_{jk} \sum_{i=1}^{S} p_{i|jk} \ln p_{i|jk}.$$

(See Appendix B for all notation.) The well-known additive decomposition for Shannon entropy is

$$H_\gamma = H_\alpha^{(1)} + \left[H_\alpha^{(2)} - H_\alpha^{(1)}\right] + \left[H_\gamma - H_\alpha^{(2)}\right] \qquad \text{(C.2)}$$

Here $H_\alpha^{(1)}$ denotes the within-population information, $\left[H_\alpha^{(2)} - H_\alpha^{(1)}\right]$ denotes the among-population information within a region, and $\left[H_\gamma - H_\alpha^{(2)}\right]$ denotes the among-region information. In the following theorem, the maximum value for each of the

latter two components is derived so that we can obtain the corresponding normalized differentiation measures.

Theorem S3.3. For the decomposition given in Eq. (C.2) in three-level hierarchy, we have the following inequalities:

$$0 \le H_\gamma - H_\alpha^{(2)} \le -\sum_{k=1}^{K} w_{+k} \ln w_{+k}; \qquad \text{(C.3)}$$

$$0 \le H_\alpha^{(2)} - H_\alpha^{(1)} \le -\sum_{k=1}^{K}\sum_{j=1}^{J_k} w_{jk} \ln \frac{w_{jk}}{w_{+k}}. \qquad \text{(C.4)}$$

When all populations have identical allele relative frequency distributions, we have $H_\gamma = H_\alpha^{(2)} = H_\alpha^{(1)}$. When all populations are completely distinct (no shared alleles), we have

$$H_\alpha^{(2)} - H_\alpha^{(1)} = -\sum_{k=1}^{K}\sum_{j=1}^{J_k} w_{jk} \ln \frac{w_{jk}}{w_{+k}} \text{ and } H_\gamma - H_\alpha^{(2)} = -\sum_{k=1}^{K} w_{+k} \ln w_{+k}.$$

Proof:

To prove Eq. (C.3), we consider the following two-level (ecosystem/region) hierarchy: there are *K* regions with allele relative frequency $p_{i|+k}$ for species *i* in region *k*, with the region weights $(w_{+1}, w_{+2}, \cdots, w_{+J})$. Note that we can express $p_{i|++}$ as

$$p_{i|++} = \sum_{k=1}^{K}\sum_{j=1}^{J_k} w_{jk} p_{i|jk} = \sum_{k=1}^{K} w_{+k} p_{i|+k}.$$

Then the "gamma" entropy for this two-level system is:

$$H_\gamma = -\sum_{i=1}^{S}\left(\sum_{k=1}^{K} w_{+k} p_{i|+k} \ln\left(\sum_{l=1}^{K} w_{+l} p_{i|+l}\right)\right).$$

The corresponding "alpha" entropy for this two-level system is $-\sum_{k=1}^{K} w_{+k} \sum_{s=1}^{S} p_{i|+k} \ln p_{i|+k}$, which is $H_\alpha^{(2)}$. Eq. (C.3) then follows directly from Theorem C.1.

To prove Eq. (C.4), we consider the following two-level hierarchy (region *k* and all populations within region *k*): there are J_k populations with allele relative frequency $p_{i|jk}$ for allele *i* in population *j*, with population weights $\left(\frac{w_{1k}}{w_{+k}}, \frac{w_{2k}}{w_{+k}}, \cdots, \frac{w_{J_k k}}{w_{+k}}\right), j = 1,2,\cdots,J_k$. The "gamma" entropy for this two-level hierarchy is the entropy value for region *k*, i.e., $H_{\alpha,k}^{(2)} = -\sum_{i=1}^{S} p_{i|+k} \ln p_{i|+k}$, where $p_{i|+k} = \sum_{j=1}^{J_k} \frac{w_{jk}}{w_{+k}} p_{i|jk}$. The corresponding "alpha" entropy is $\sum_{j=1}^{J_k} \frac{w_{jk}}{w_{+k}} H_{\alpha,jk}^{(1)} = -\sum_{j=1}^{J_k} \frac{w_{jk}}{w_{+k}} \sum_{i=1}^{S} p_{i|jk} \ln p_{i|jk}$. Then Theorem C.1 leads to

$$H_{\alpha,k}^{(2)} - \sum_{j=1}^{J_k} \frac{w_{jk}}{w_{+k}} H_{\alpha,jk}^{(1)} = -\sum_{i=1}^{S} p_{i|+k} \ln p_{i|+k} + \sum_{j=1}^{J_k} \frac{w_{jk}}{w_{+k}} \sum_{i=1}^{S} p_{i|jk} \ln p_{i|jk} \le -\sum_{j=1}^{J_k} \frac{w_{jk}}{w_{+k}} \ln \frac{w_{jk}}{w_{+k}}.$$

Summing over *k* with weight w_{+k}in both sides of the above inequality, we obtain

$$-\sum_{k=1}^{K} w_{+k} \sum_{i=1}^{S} p_{i|+k} \ln p_{i|+k} + \sum_{k=1}^{K}\sum_{j=1}^{J_k} w_{jk} \sum_{i=1}^{S} p_{i|jk} \ln p_{i|jk} = H_\alpha^{(2)} - H_\alpha^{(1)} \le -\sum_{j=1}^{J_k} w_{jk} \ln \frac{w_{jk}}{w_{+k}}.$$

This proves Eq. (C.4).

From the above theorem, we have $0 \le H_\alpha^{(1)} \le H_\alpha^{(2)} \le H_\gamma$, i.e., the gamma diversity of any level is greater than or equal to the corresponding alpha diversity at the same level. Eq. (C.3) leads to the following normalized differentiation measure among regions:

$$\Delta_D^{(2)} = \frac{H_\gamma - H_\alpha^{(2)}}{-\sum_{k=1}^{K} w_{+k} \ln w_{+k}}. \qquad \text{(C.5)}$$

Likewise, Eq. (C.4) leads to the following normalized differentiation measure among populations within a region:

$$\Delta_D^{(1)} = \frac{H_\alpha^{(2)} - H_\alpha^{(1)}}{-\sum_{k=1}^{K}\sum_{j=1}^{J_k} w_{jk}\ln(w_{jk}/w_{+k})}. \qquad \text{(C.6)}$$

Each of the two differentiation measures takes the minimum value of 0 when all populations have identical allele relative frequency distributions and it takes the maximum value of 1 when all populations are completely distinct (no shared species). Note that in the latter case, we can decompose the gamma diversity as

$$H_\gamma = -\sum_{i=1}^{S}\left(\sum_{k=1}^{K}\sum_{j=1}^{J_k} w_{jk}p_{i|jk}\ln(w_{jk}p_{i|jk})\right)$$

$$= -\sum_{i=1}^{S}\left(\sum_{k=1}^{K}\sum_{j=1}^{J_k} w_{jk}p_{i|jk}\left(\ln p_{i|jk} + \ln\frac{w_{jk}}{w_{+k}} + \ln w_{+k}\right)\right)$$

$$= -\sum_{i=1}^{S}\left(\sum_{k=1}^{K}\sum_{j=1}^{J_k} w_{jk}p_{i|jk}\ln p_{i|jk}\right) - \sum_{i=1}^{S}\left(\sum_{k=1}^{K}\sum_{j=1}^{J_k} w_{jk}p_{i|jk}\ln\frac{w_{jk}}{w_{+k}}\right)$$

$$-\sum_{i=1}^{S}\left(\sum_{k=1}^{K}\sum_{j=1}^{J_k} w_{jk}p_{i|jk}\ln w_{+k}\right)$$

$$= H_\alpha^{(1)} - \sum_{k=1}^{K}\sum_{j=1}^{J_k} w_{jk}\ln\frac{w_{jk}}{w_{+k}} - \sum_{k=1}^{K} w_{+k}\ln w_{+k}$$

$$\equiv H_\alpha^{(1)} + \left[H_\alpha^{(2)} - H_\alpha^{(1)}\right] + \left[H_\gamma - H_\alpha^{(2)}\right].$$

In this special case, we have $H_\alpha^{(2)} - H_\alpha^{(1)} = -\sum_{k=1}^{K}\sum_{j=1}^{J_k} w_{jk}\ln\frac{w_{jk}}{w_{+k}}$ and $H_\gamma - H_\alpha^{(2)} = -\sum_{k=1}^{K} w_{+k}\ln w_{+k}$.

As we proved in Theorem C.3, each differentiation measure can be regarded as based on a two-level hierarchy. Thus, each differentiation satisfies the corresponding monotonicity and "true dissimilarity" properties stated in Theorem C.2.

Phylogenetic differentiation measures in three-level hierarchy

For phylogenetic differentiation measures based on ultrametric trees, all derivation steps are parallel to those of allelic diversity. Consider the three-level hierarchy (ecosystem-region-population) in Table 1 of the main text. Shannon gamma and alpha entropies are expressed as (Table 3 of the main text):

$I_\gamma = -\sum_{i=1}^{B} L_i a_{i|++}\ln a_{i|++}$, $I_\alpha^{(2)} = -\sum_{k=1}^{K} w_{+k}\sum_{i=1}^{B} L_i a_{i|+k}\ln a_{i|+k}$,

$I_\alpha^{(1)} = -\sum_{k=1}^{K}\sum_{j=1}^{J_k} w_{jk}\sum_{i=1}^{B} L_i p_{i|jk}\ln p_{i|jk}$.

Corresponding to Eq. (C.2), we have a similar decomposition for phylogenetic entropy:

$$I_\gamma = I_\alpha^{(1)} + \left[I_\alpha^{(2)} - I_\alpha^{(1)}\right] + \left[I_\gamma - I_\alpha^{(2)}\right] \qquad \text{(C.7)}$$

Here, $I_\alpha^{(1)}$ denotes the within-population phylogenetic information, $\left[I_\alpha^{(2)} - I_\alpha^{(1)}\right]$ denotes the among-population phylogenetic information within a region, and $\left[I_\gamma - I_\alpha^{(2)}\right]$ denotes the among-region phylogenetic information. The maximum value for each of the latter two components is derived in the following theorem so that we can obtain the corresponding differentiation measures.

<u>Theorem C.4.</u> For the decomposition given in Eq. (C.7) in the three-level hierarchy, we have the following inequalities under an ultrametric tree with depth *T*:

$$0 \le I_\gamma - I_\alpha^{(2)} \le -T \sum_{k=1}^{K} w_{+k} \ln w_{+k}; \tag{C.8}$$

$$0 \le I_\alpha^{(2)} - I_\alpha^{(1)} \le -T \sum_{k=1}^{K} \sum_{j=1}^{J_k} w_{jk} \ln \frac{w_{jk}}{w_{+k}}. \tag{C.9}$$

When all populations have identical allele relative frequency distributions, we have $I_\gamma = I_\alpha^{(2)} = I_\alpha^{(1)}$. When all populations are completely distinct phylogenetically (no shared branches across populations, though branches within a population may be shared), we have $I_\alpha^{(2)} - I_\alpha^{(1)} = -T \sum_{k=1}^{K} \sum_{j=1}^{J_k} w_{jk} \ln \frac{w_{jk}}{w_{+k}}$ and $I_\gamma - I_\alpha^{(2)} = -T \sum_{k=1}^{K} w_{+k} \ln w_{+k}$.

The above theorem leads to the two normalized phylogenetic differentiation measures (given in Table 3 of the main text) in the range [0, 1]. Each of the two phylogenetic differentiation measures takes the minimum value of 0 when all populations have identical allele relative frequency distributions and it takes the maximum value of 1 when all populations are phylogenetically completely distinct. All the derivation procedures as well as the monotonicity and “true dissimilarity” properties are parallel to those of allelic diversity and thus are omitted.
